# MutMapPlus identified novel mutant alleles of a rice *starch branching enzyme IIb* gene for fine‐tuning of cooked rice texture

**DOI:** 10.1111/pbi.12753

**Published:** 2017-06-14

**Authors:** Masaru Nakata, Tomomi Miyashita, Rieko Kimura, Yuriko Nakata, Hiroki Takagi, Masaharu Kuroda, Takeshi Yamaguchi, Takayuki Umemoto, Hiromoto Yamakawa

**Affiliations:** ^1^ Division of Crop Development Central Region Agricultural Research Center National Agriculture and Food Research Organization (NARO) Joetsu Japan; ^2^ Department of Bioproduction Science Ishikawa Prefectural University Nonoichi Japan; ^3^ Institute of Crop Science National Agriculture and Food Research Organization (NARO) Tsukuba Japan

**Keywords:** amylopectin, cooked rice texture, mutant allele, MutMapPlus, *Oryza sativa*, starch branching enzyme

## Abstract

Physicochemical properties of storage starch largely determine rice grain quality and food characteristics. Therefore, modification of starch property is effective to fine‐tune cooked rice textures. To obtain new resources with modified starch property as breeding materials, we screened a mutant population of a *japonica* cultivar Nipponbare and found two independent mutant lines, *altered gelatinization* (*age*)*1* and *age2*, with moderate changes in starch gelatinization property. A combination of conventional genetic analyses and the latest mapping method, MutMapPlus, revealed that both of these lines harbour novel independent mutant alleles of *starch branching enzyme IIb* (*BEIIb*) gene. In *age1*, amino acid substitution of Met‐723 to Lys completely abolished BEIIb enzyme activity without significant reduction in its protein level. A transposon insertion in an intron of *BEIIb* gene reduced BEIIb protein level and activity in *age2*. Production of a series of the mutant lines by combining *age* alleles and *indica*‐type *starch synthase IIa* allele established stepwise alteration of the physicochemical properties of starch including apparent amylose content, thermal property, digestibility by α‐amylase and branched structures of amylopectin. Consistent with the alteration of starch properties, the results of a sensory evaluation test demonstrated that warm cooked rice of the mutants showed a variety of textures without marked reduction in overall palatability. These results suggest that a series of the mutant lines are capable of manipulation of cooked rice textures.

## Introduction

Starch constitutes most of the weight of cereal grains and is the most important carbohydrate glucose polymer for humans as a dietary source of energy. Starch consists of linear or slightly branched amylose and highly branched amylopectin. The amylose content and chain length distribution (CLD) of amylopectin branches are the predominant determinant of the physicochemical properties of starch and the cooking properties of grains.

Starch is synthesized by the orchestrated functional interactions of four classes of enzymes, ADP‐glucose pyrophosphorylase, starch synthase (SS), starch branching enzyme (BE) and starch debranching enzyme (DBE) (Ball and Morell, 2003; Nakamura, 2002; Smith *et al*., 1997; Zeeman *et al*., 2010). Of these, SS elongates the nonreducing end of α‐1,4‐glucan chains of amylose and amylopectin by the addition of glucose from ADP‐glucose, while BE introduces α‐1,6‐branched glucosidic linkages into α‐1,4‐glucan chains. These activities are essential for the synthesis of the branched structure of amylopectin.

In rice, SSI, SSIIa and SSIIIa are highly expressed in developing endosperm and elongate very short (degree of polymerization (DP) 6 and 7), short (DP 6–10) and long (DP > 33) amylopectin chains, respectively (Fujita *et al*., 2006, 2007; Nakamura *et al*., 2005; Ohdan *et al*., 2005). Two groups of rice varieties, *indica* and *japonica*, harbour different *SSIIa* alleles (referred to as *SSIIa* ^*indica*^ and *SSIIa* ^*japonica*^, respectively), which produce active and inactive SSIIa, respectively (Umemoto *et al*., 2004). Such a difference in the *SSIIa* alleles alters the structure of amylopectin, of which intermediate chains (DP 12–24) are enriched, and short chains (DP < 11) are depleted in *indica* varieties compared to *japonica* varieties (Umemoto *et al*., 2002). Four amino acid substitutions were found between *SSIIa* ^*japonica*^ and *SSIIa* ^*indica*^ alleles, and two of them were identified as indispensable amino acids for full SS activity (Bao *et al*., 2006; Nakamura *et al*., 2005; Umemoto and Aoki, 2005; Waters *et al*., 2006).

Three BE isoforms, *BEI*,* BEIIa* and *BEIIb*, are encoded in the rice genome. *In vitro* biochemical analysis revealed that BEIIb exclusively transfers short chains (DP 6 and 7), and BEIIa has a more extensive transfer preference (DP 6–15), while BEI transfers intermediate chains (DP 26–39) in addition to short chains (DP 6–12) (Nakamura *et al*., 2010). Compared to BEI‐ and BEIIa‐deficient mutants, several BEIIb‐deficient mutants, referred to as *amylose extender* (*ae*), exhibit severe effects on grain appearance and starch properties (Kubo *et al*., 2010; Nishi *et al*., 2001; Satoh *et al*., 2003a,b; Tanaka *et al*., 2004). Starch of an *ae* mutant, EM10, showed about 13 °C higher peak gelatinization temperature (*T*_p_) compared to the original cultivar Kinmaze (Tanaka *et al*., 2004). Elevation of gelatinization temperature in EM10 is consistent with the structure of amylopectin, in which short chains (DP 6–17) and intermediate to long chains (DP > 18) were markedly decreased and increased, respectively. Complementation experiments of EM10 revealed that altered starch properties were restored by introducing functional BEIIb driven by its own promoter and that the degree of the restoration depended on the level of BEIIb activity (Tanaka *et al*., 2004). This suggests that control of BEIIb activity is effective to manipulate the starch properties.

Among recent breeding programmes of rice grain quality, variation of cooked rice texture without impairment of overall palatability is expected. For example, harder and less sticky rice cultivars can avoid excessive adhesion of cooked materials onto containers, and thus improve handling efficiency during packaging in food industry. To achieve this requirement, development of a series of lines showing a stepwise difference in starch property is demanded. A combination of mutant alleles for starch synthesizing enzymes that specifically function in endosperm, such as BEIIb and SSIIa, would be good candidates to modify characteristics of storage starch. As alleles with severe defects in the functions of the enzymes often lead to an excess in the effects on starch properties and profound reduction in palatability [e.g. assumed reasonable utilizations of EM10 are not cooked rice but food industrial materials (Takahashi *et al*., 2001)], the isolation of alleles with modest effects is desired.

Upon conventional genetic mapping procedure, repeated evaluation of a phenotype of interest in a large number of segregating populations until identification of the causal gene/mutation is a labour‐intensive and time‐consuming process. Recently developed novel genetic mapping procedures using high‐throughput next‐generation DNA sequencers (NGS) solved the bottleneck by greatly reducing the number of times of the phenotyping process and accelerated identification of the causal mutation governing the agronomic trait. MutMap is one of the procedures and is based on a crossing of the mutant to the original parental line used for mutagenesis, followed by resequencing of the genome DNA of a bulk of F2 segregants with mutant phenotypes obtained by self‐pollination of F1 (Abe *et al*., 2012). Takagi *et al*. (2015) applied MutMap to identify a mutation related to salt‐tolerance and verified the advantage of the procedure by production of a high salt‐tolerant rice cultivar within a few years. However, MutMap requires an original parental line to build the high‐quality reference genome and to eliminate background false‐positive SNPs, although in many mutant populations such parental line has not been available. Furthermore, MutMap cannot be applied to mutants that are not amenable for crossing. To address these fundamental issues of MutMap, an advanced method, MutMapPlus, in which whole‐genome sequences between two sibling bulks of mutant (M) and wild‐type (WT) segregants were compared to identify causal mutations, was developed (Fekih *et al*., 2013).

In this study, to provide novel breeding resources with linked DNA markers for fine‐tuning cooked rice textures, we screened the mutant lines exhibiting altered grain starch properties and identified two novel mutant alleles of *BEIIb* designated *altered gelatinization*s (*age*s) by a combination of the conventional mapping and MutMapPlus procedures. A point mutation of *BEIIb* gene in *age1* allele caused BEIIb‐deficient phenotype, which is permissive for cooked rice even though there was a complete disappearance of its enzyme activity. In addition, a transposon insertion into the same gene yielded another *age* allele, named *age2*, resulting in milder modification of the activity. Both *age* alleles exhibited moderate effects on cooked rice textures as well as the physicochemical properties of their starch. Our work would not only provide breeding materials for fine‐tuning cooked rice textures and confirm the general versatility of MutMapPlus but also shed novel insights into the regulation of BEIIb functions in starch synthesis.

## Results

### Isolation of novel mutant lines with altered gelatinization properties

**Figure 1:**
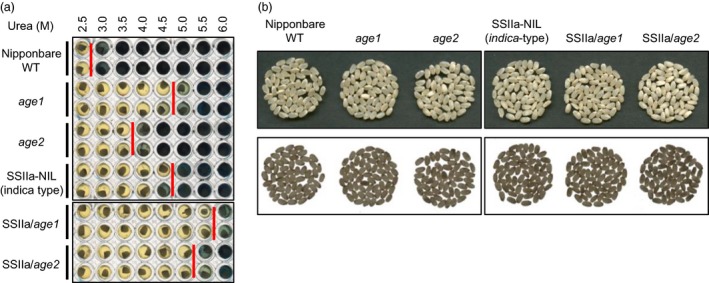
Gelatinization properties and grain appearance of *age1*,* age2*, SSIIa‐NIL and their double allele lines. (a) Gelatinization of endosperm starch at various concentrations of urea solution. Urea concentration of onset gelatinization was shown by red bars. (b) Grain appearance. Images by reflected light (upper panel) and transmitted light (lower panel) are shown. Parent plants of the seeds used in this experiment were grown in the paddy field of Hokuriku Research Station (Joetsu, Japan) in 2016.

We screened a Nipponbare mutant population by evaluation of solubility of endosperm starch in 3.6 m urea solution, in which Nipponbare starch is gelatinized. Among the 3300 mutant lines examined, we found two recessive mutant lines whose starches were hardly gelatinized in the urea solution (Figure 1a). Starches from these lines were gelatinized by urea solution at different concentrations, suggesting that these lines harbour independent alleles affecting the gelatinization property of starch. Alleles with severe and moderate effects on starch gelatinization were referred to as *age1* and *age2*, respectively. The grain appearance of *age* lines was almost normal, and a small proportion of white core grains was observed (Figure 1b).

**Figure 2:**
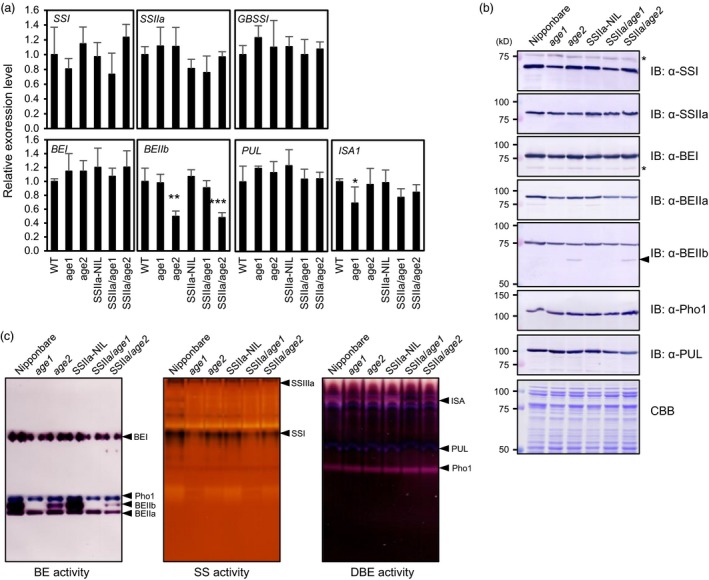
Expression level and enzyme activity of starch biosynthetic enzymes. (a) mRNA levels of starch biosynthetic enzyme genes in developing caryopses were analysed by qRT‐PCR. *eEF‐1*α was used as an internal control. The value for Nipponbare (WT) was set to 1, and relative values were shown. Asterisks indicate significant difference from background lines (Nipponbare or SSIIa‐NIL) of each line (Student's *t* test, *P <* *0.05, **P <* *0.01, ***P <* *0.001). (b) Immunoblot (IB) analysis of soluble endosperm extract from developing seeds. Arrowhead on the right indicates bands corresponding to truncated BEIIb. Asterisks indicate nonspecific signals. (c) branching enzyme, starch synthase and debranching enzyme activities in soluble extract of developing endosperm were detected by activity staining after native‐PAGE. Enzymes corresponding to each activity band were indicated on the right in each panel.

The expression level and enzyme activity of several starch biosynthetic enzymes in developing caryopses were examined by quantitative RT‐PCR (qRT‐PCR), immunoblot (IB) and activity staining analyses. *ISA1* and *BEIIb* mRNA levels were significantly down‐regulated in *age1* and *age2*, respectively (Figure 2a). Despite a slight reduction of BEIIb protein in *age1* (76% of Nipponbare), its enzyme activity was completely abolished (Figure 2b,c). In *age2*, the BEIIb protein level (67% of Nipponbare) and its enzyme activity were lower than that of Nipponbare. An additional smaller molecular weight protein was detected in *age2* (Figure 2b). We also analysed starch‐binding ability of starch biosynthetic enzymes. SSIIa^indica^ showed strong binding to starch granules as reported by Umemoto and Aoki (2005) (Figure S1). Slight increase in SSIIa, BEI, BEIIa and BEIIb in *age1* was also observed. These results suggest that the altered gelatinization property of *age1* and *age2* is related to the alteration of BEIIb activity.

### Identification of causal mutation of *age1* by a combination of conventional mapping and MutMapPlus

**Figure 3:**
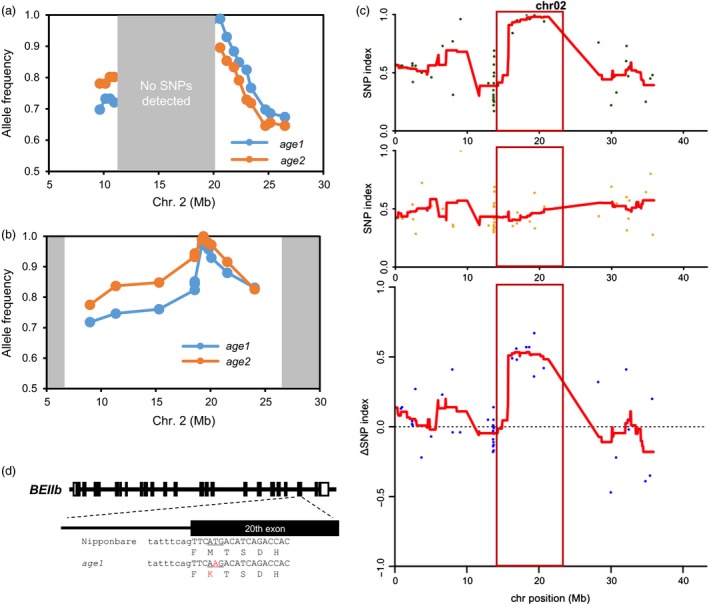
Genetic mapping of *age1*. (a) and (b) Nipponbare‐type allele frequency plot of chromosome 2 generated by rough mappings. The results of *age* mutants‐Koshihikari (a) and *age* mutants‐SL09 (b) F2 populations are shown. The regions in which no polymorphisms were detected are shaded with grey boxes. (c) SNP index and Δ(SNP index) plots of the chromosome 2 generated by MutMapPlus analysis. SNP index of the mutant (M) bulk (upper panel) and the wild‐type (WT) bulk (middle panel) and Δ(SNP index) calculated by subtraction of the index of WT bulk from that of M bulk (lower panel) is shown. Red lines indicate the sliding window average of 4 Mb intervals with 50 kb increment. Candidate region confined by the Fisher's *P* value of <0.05 is indicated with a red box. (d) Schematic diagrams of *BEIIb* gene in *age1* allele. Open and black boxes indicate untranslated regions and exons, respectively. Uppercase and lowercase letters indicate DNA sequence of exon and intron, respectively. Translated amino acids are shown under the DNA sequence. Substituted nucleotide and amino acid are shown by red letter.

Rough mapping was performed by using F2 populations derived from the crosses of each *age* line and another *japonica* cultivar, Koshihikari. Illumina single‐nucleotide polymorphism (SNP) array analysis indicated that the mutated locus in both *age1* and *age2* was mapped within the region between 11 and 21 Mb on chromosome 2 (Figure 3a). However, as the region is common among Nipponbare and Koshihikari (Yamamoto *et al*., 2010) and no SNP was detected in the region, we could not narrow down the genomic region corresponding to *age* phenotype. Then, further mapping was conducted with F2 progenies generated by crosses of each *age* line and SL09, which have a chromosome segment of an *indica* cultivar, Kasalath, between 6.6 and 27.1 Mb of chromosome 2 on Nipponbare background genome, so that the effect of another gelatinization‐controlling allele, *SSIIa* ^*indica*^, which is located on chromosome 6, was eliminated. The mapping using SSR markers focused both *age* mutations between 19.2 and 19.8 Mb (Figure 3b), presuming that the causal gene is *BEIIb*, which resides at 19.35 Mb of the chromosome.

In order to exclude the possibility that the *age* phenotypes are attributed to mutations of closely linked neighbouring genes other than *BEIIb*, we next employed MutMapPlus for identification of a corresponding mutation. We applied this method to the F2 progenies of *age1*‐Nipponbare and *age2*‐Nipponbare crosses with a modification to detect all types of SNPs, as the original MutMapPlus algorithm extracted only transition‐type SNPs. For the analysis of *age1*, 54 802 734 and 54 196 761 aligned reads of M and WT bulks, corresponding to 21.1 and 20.9 times coverage of the rice genome, respectively, drew similar charts of SNP index, with an exception that a unique peak was detected only for the M bulk at 15–25 Mb on chromosome 2, and the Δ(SNP index) was also peaked at the same location (the expected Δ(SNP index) is 0.67) (Figure 3c, Figure S2). Within the candidate region confined by the Fisher's *P* values lower than 0.05, corresponding to 13.75–23.3 Mb, we extracted eight SNPs, that nearly agreed with the expected SNP indexes, 1 and 0.33 for M and WT bulks, respectively (Table 1). Among them, the most likely candidate SNP (19 357 116 base) was located at the 20th exon of *BEIIb* gene that leads to nonsynonymous substitution of Met‐723 (from translation start site) to Lys (M723K) (Figure 3d, Table 1). The other seven SNPs were located at intergenic regions, although one mutation of 18 242 584 base seemed to reside in a promoter region of a hypothetical gene.

**Table 1:** Single‐nucleotide polymorphisms (SNPs) within the candidate genomic region identified by MutMapPlus analysis of *age1*

Chromosome	Base	Nipponbare reference base	Mutant bulk			Wild‐type bulk			Fisher's *P* value	Annotation
			Base	Number of reads	SNP index	Base	Number of reads	SNP index		
chr02	16 289 640	C	Y	19	0.84	Y	17	0.35	0.005379	Intergenic.
chr02	16 865 416	A	G	19	0.94	R	16	0.38	0.000556	Intergenic.
chr02	16 929 731	A	T	23	0.95	W	32	0.47	0.000111	Intergenic.
chr02	18 242 584	C	T	25	1.00	Y	23	0.43	0.000006	110 bp upstream of Hypothetical protein (Os02g0510400) gene.
chr02	18 648 446	G	A	26	0.96	R	23	0.39	0.000019	Intergenic.
chr02	19 320 101	G	C	26	1.00	S	11	0.64	0.004997	Intergenic.
chr02	19 357 116	A	T	18	1.00	W	27	0.33	0.000004	Starch branching enzyme, *BEIIb* (Os02g0528200), M723K.
chr02	20 670 926	T	A	18	0.94	W	27	0.52	0.002882	Intergenic.

**Figure 4:**
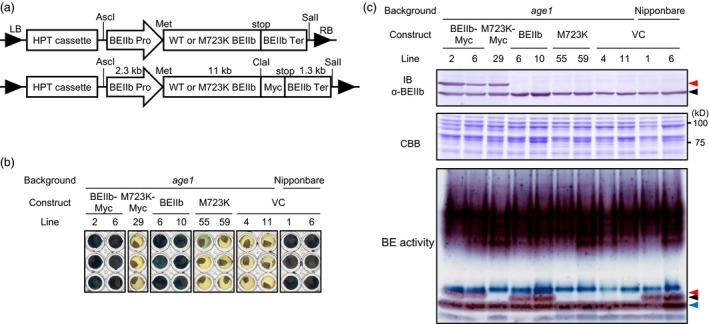
Complementation of *age1* by genomic fragment of *BEIIb* gene. (a) Schematic diagrams of constructs used for complementation. LB, left border; RB, right border; HPT, hygromycin phosphotransferase; Pro, promoter; Ter, terminator; Met, translation start site; stop, translation termination site. Restriction sites were indicated above. (b) Gelatinization of endosperm starch of complementation lines in 5.0 m urea solution. VC, vector control. (c) IB analysis using anti‐BEIIb antibody (upper panel), CBB staining (middle panel) and branching enzyme activity staining (lower panel) of soluble extract of developing endosperm. Black, red and blue arrowheads indicate bands corresponding to BEIIb, Myc‐tagged BEIIb and BEIIa, respectively. The transgenic plants were grown in a greenhouse.

To confirm M723K substitution of BEIIb is the causal mutation of *age1* phenotype, constructs consisting of a genomic fragment of WT or M723K mutant‐type *BEIIb* gene including the 2.4 kb upstream region from the translation start site were introduced into *age1* (Figure 4a). Constructs containing four times‐repeated Myc epitope tags at the carboxy (C)‐terminal of BEIIb were also introduced. The endosperm starch of BEIIb‐ or BEIIb‐Myc‐expressing lines was gelatinized in 5.0 m urea solution, in which vector control seeds of Nipponbare background but not *age1* background were gelatinized, indicating successful restoration of *age1* phenotype (Figure 4b, Figure S3). In contrast, the expression of M723K or M723K‐Myc failed to restore the gelatinization property of *age1* (Figure 4b). The enzyme activity of BEIIb was detected in BEIIb‐ or BEIIb‐Myc‐expressing lines but not in M723K‐ or M723K‐Myc‐expressing lines (Figure 4c). The difference in urea concentration for gelatinization in this experiment from that shown in Figure 1a would arise from the difference in ripening conditions between the paddy field and the greenhouse, as the ripening temperature affects the crystallinity of synthesized starch. These lines of evidence indicate that M723K mutation in BEIIb eliminates its enzyme activity and is the causal mutation of *age1* phenotype.

### *Tos17* insertion in *BEIIb* gene in *age2*

**Figure 5:**
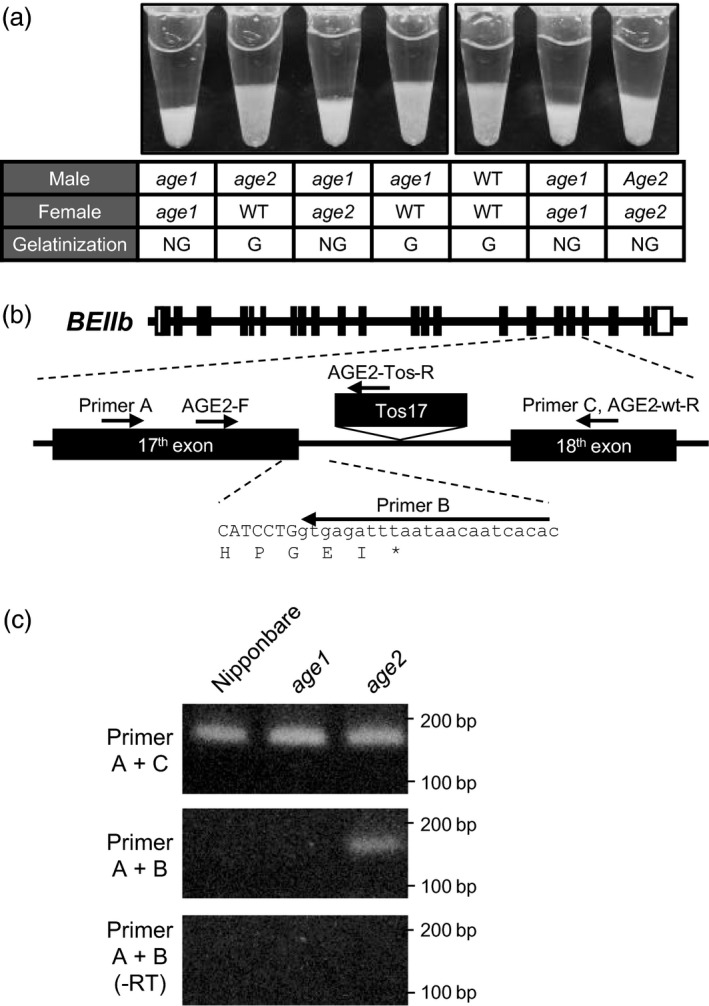
Insertion of *Tos17* in *BEIIb* gene in *age2*. (a) Gelatinization of endosperm starch of F1 seeds in 5.0 m urea solution. Lines used for each cross and evaluation of gelatinization were shown under the image. WT, G and NG indicate Nipponbare, gelatinized and not gelatinized, respectively. (b) Schematic diagrams of *BEIIb* gene in *age2*. Primers used in (c) and Figure 6 were shown by arrows. Open and black boxes indicate untranslated regions and exons, respectively. Location of *Tos17* insertion is indicated above the gene. Uppercase and lowercase letters indicate DNA sequence of exon and intron, respectively. Translated amino acids were shown under the DNA sequence. (c) Alternatively spliced *BEIIb* transcript in *age2* was detected by RT‐PCR analysis. Total RNA without reverse transcription (‐RT) was used as a template for negative control (lower panel). DNA size markers are indicated on the right.

To analyse allelism between *age1* and *age2*, they were crossed, and gelatinization of the endosperm starch of F1 grains by urea was determined by the volume of sediment (Figure 5a, Figure S4). Starches obtained by crossing *age1* and *age2* were not gelatinized in 5.0 m urea solution, while those obtained by crossing Nipponbare and *age1* or *age2* were gelatinized, indicating that *age1* and *age2* are allelic. Sequence analysis of *BEIIb* gene in *age2* found an insertion of a retrotransposon, *Tos17*, in the 17th intron of *BEIIb* gene (Figure 5b). Application of MutMapPlus to *age2* failed to detect functional SNPs, but the survey of short reads mapped around *BEIIb* indicated that insertion of an interrupting sequence at the position described above was found in M bulk (data not shown). Furthermore, whole‐genome resequencing data confirmed that no SNPs introducing amino acid substitutions were found within the region from 10 Mb to the end of chromosome 2 in *age2* (Table S1).

The smaller molecular weight‐immunoreacted protein in *age2* endosperm extract in Figure 2b is estimated to be a truncated form of BEIIb, because the *Tos17* insertion likely leads to aberrant splicing in the 17th exon–intron junction and introduces a premature stop codon in the *BEIIb* transcripts (Figure 5b). Indeed, a 17th intron‐containing *BEIIb* transcript as well as a correctly spliced one was produced in *age2* (Figure 5c). Migration of the truncated BEIIb corresponds to its estimated molecular weight (approximately 67 kD) in comparison with that of intact mature BEIIb (approximately 86 kD) (Figure 2c). Therefore, the reduction of full‐length *BEIIb* transcript in *age2* by occasional mis‐splicing caused by a *Tos17* insertion leads to a weak BEIIb‐deficient phenotype. These results clearly illustrate the different severities of the altered gelatinization phenotype between *age1* and *age2*.

### Development of DNA markers for detection of *age1* and *age2* alleles

**Figure 6:**
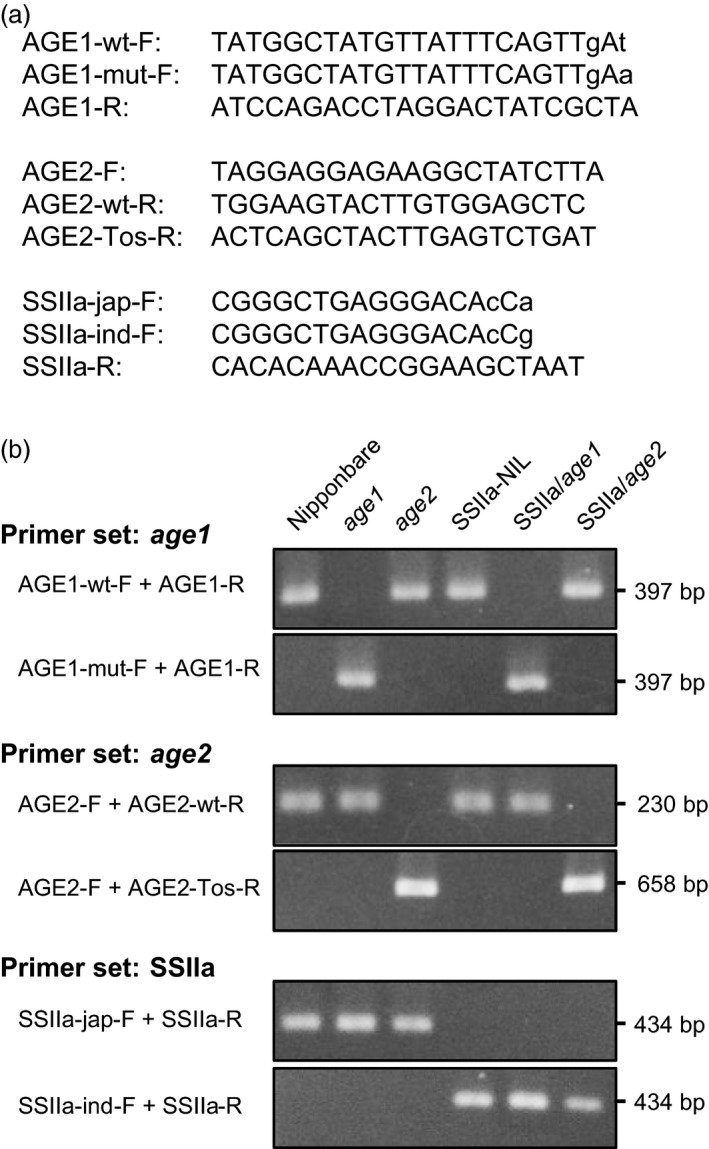
DNA markers for detection of *age1* and *age2* alleles and *SSIIa* ^*indica*^ allele. (a) Primers for DNA markers. DNA sequence shown is 5′ to 3′. Allele‐specific and additional mismatch nucleotides are shown in lowercase letters. (b) Band patterns of amplified DNA. Amplicon lengths are indicated on the right.

We developed a PCR‐based SNP marker for detection of *age1* allele. Two forward primers whose 3′ ends locate to the position of SNP (one is specific to *WT* allele and the other is specific to *age1* allele) were designed with an additional mismatch introduced in the third nucleotide from the 3′ end, while a reverse primer is common (Figure 6a). These specific primer sets exclusively amplified *WT* or *age1* alleles, respectively (Figure 6b).

For *age2*, a common forward primer and WT‐specific reverse primer were designed on the 17th exon and the 18th exon, respectively, for detection of *WT* allele (Figures 5b and 6a). Another reverse primer was designed on *Tos17*. A combination of the reverse primer and common forward primer specifically amplified a DNA fragment composed of a part of the *BEIIb* genomic region and *Tos17* flanking region.

These DNA markers established strict discrimination of *WT* and *age* alleles of *BEIIb* gene.

### Introgression of *SSIIa* ^*indica*^ allele into *age1* and *age2*

To obtain further variation in the starch gelatinization property, we crossed *age1* and *age2* with Nipponbare near‐isogenic line (NIL) harbouring *SSIIa* ^*indica*^ allele (Umemoto *et al*., 2004). A PCR‐based SNP marker to distinguish *SSIIa* ^*indica*^ and *SSIIa* ^*japonica*^ alleles was designed based on the primer set for SNP3 (Met/Val‐737 in *japonica*/*indica*) (Hiratsuka *et al*., 2010) with slight modification to optimize to our PCR condition (Figure 6). Plants homozygous for *age1* or *age2* allele and *SSIIa* ^*indica*^ allele were obtained by the DNA marker‐assisted selection and designated as SSIIa/*age1* or SSIIa/*age2*, respectively. Starches of the double allele lines were gelatinized in urea solution at a higher concentration compared to their parental *age* lines (Figure 1a). The expression level and activity of BEIIb in the double allele lines were comparable to those in each parental *age* line (Figure 2). These results indicate that the increased severity of the gelatinization property in the double allele lines arises from a combination of impairment of BEIIb and active SSIIa. We could not identify the SSIIa activity band in SSIIa‐NIL extract by SS activity staining (Figure 2c) because SSIIa activity is weak and hardly detected by this staining method as observed previously (Umemoto *et al*., 2004).

### Grain size and starch parameters

The grain weight of *age1*, SSIIa/*age1* and SSIIa/*age2* was reduced (Table 2). It is considered that reduction of grain weight in *age1* and SSIIa/*age2* originates from reduced thickness of the grains, while SSIIa/*age1* reduced grain weight without a significant change of grain size. The effect of *age* alleles on the grain weight was quite weak compared to EM10, whose grain weight was about 65% of that of Kinmaze (Nishi *et al*., 2001).

**Table 2:** Grain size, apparent amylose content and thermal properties of starch

	Weight (mg)^a^	Length (mm)^b^	Width (mm)^b^	Thickness (mm)^b^	Amylose content (%)^c^	Gelatinization temperature (°C)^d^		
						*T*_o_	*T*_p_	*T*_c_
Nipponbare	22.14 ± 0.35 (100)	5.16 ± 0.03	2.83 ± 0.09	2.07 ± 0.02	21.5 ± 0.9	63.4 ± 0.2	75.3 ± 0.2	69.8 ± 0.1
*age1*	19.49 ± 1.50* (88)	5.18 ± 0.09	2.84 ± 0.03	1.96 ± 0.05*	29.4 ± 0.4***	71.5 ± 0.6***	85.3 ± 0.4***	79.5 ± 0.5***
*age2*	22.37 ± 0.55 (101)	5.38 ± 0.00***	2.87 ± 0.03	2.07 ± 0.02	22.9 ± 0.2*	68.4 ± 0.3***	81.3 ± 0.4***	74.2 ± 0.3***
SSIIa‐NIL	21.50 ± 0.57 (97)	5.33 ± 0.08*	2.88 ± 0.03	2.09 ± 0.02	17.7 ± 0.5**	72.2 ± 0.0***	80.5 ± 0.2***	76.5 ± 0.1***
SSIIa/*age1*	19.78 ± 0.37** (89)	5.28 ± 0.01**	2.77 ± 0.04	2.05 ± 0.03	28.8 ± 0.5***	79.2 ± 0.5***	89.4 ± 1.2***	84.7 ± 0.2***
SSIIa/*age2*	20.41 ± 0.24** (92)	5.32 ± 0.13	2.82 ± 0.05	2.01 ± 0.01*	24.4 ± 0.2**	76.4 ± 0.4***	89.2 ± 0.7***	83.7 ± 0.5***

a Weight is mean ± SD of at least three independent sets of measurement of 40–70 mature brown rice. Percentages of the weight relative to Nipponbare weight were shown in parentheses.

b Length, width and thickness are mean ± SD of three independent sets of measurement of five mature brown rice.

c Values are the means ± SD (*n* = 3).

d *T*_o_, *T*_p_ and *T*_c_ indicate onset, peak and conclusion temperatures, respectively.

Asterisks indicate significant difference from Nipponbare (Student's *t* test, *P <* *0.05, **P <* *0.01, ***P <* *0.001).

**Figure 7:**
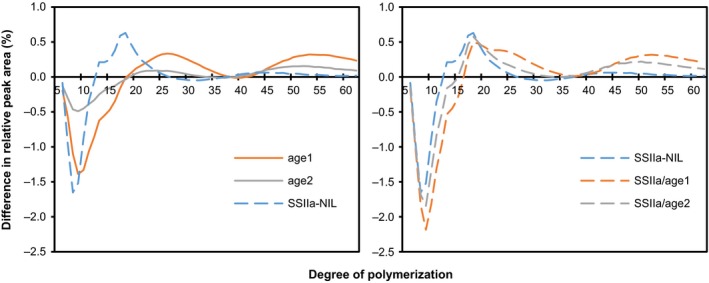
Chain length distribution (CLD) patterns of amylopectin. CLD of amylopectin was examined by HPAEC‐PAD, and then the difference in the profiles between each line and Nipponbare was calculated by subtracting the ratio of a chain of given length of the sample with that of the mean value of Nipponbare. Values are the means of three replicates. The grains used for this experiment were ripened in the paddy field.

Compared to Nipponbare (21.5%), *age1* and *age2* lines showed increased apparent amylose content to around 30% and 23%, respectively (Table 2). This is consistent with the results of *ae* mutants (Nakamura *et al*., 2011; Nishi *et al*., 2001). Although the apparent amylose content of SSIIa‐NIL was significantly lower than Nipponbare, SSIIa/*age1* and SSIIa/*age2* showed almost the same apparent amylose content as *age1* and *age2*, respectively. The increase of apparent amylose content in the mutant lines is, at least in part, likely to be due to the altered CLD of amylopectin as described below (Figure 7).

The thermal properties of purified starch were analysed by differential scanning calorimetry. *T*_p_ in *age1* and *age2* lines was 10 °C and 6 °C higher than that of Nipponbare, respectively (Table 2). SSIIa‐NIL also exhibited a significantly (around 5 °C) higher *T*_p_ value than Nipponbare, and SSIIa/*age1* and SSIIa/*age2* showed a further increase in it. Particularly, *T*_p_ of SSIIa/*age2* was comparable to that of SSIIa/*age1* (14 °C higher than Nipponbare), suggesting that a combination of *SSIIa* ^*indica*^ and *age2* alleles provided an additive or even transcending effect on the elevation of *T*_p_.

### Fine structure of amylopectin

The CLD of amylopectin was examined by high‐performance anion‐exchange chromatography‐pulsed amperometric detection (HPAEC‐PAD) (Figure 7). Amylopectin in *age1* and *age2* showed similar CLD patterns, in which the proportion of short chains (DP 6–17) was significantly reduced, and that of intermediate (DP 19–33) and long chains (DP > 41) was increased. Consistent with BEIIb activity (Figure 2c), the extent of the alteration was markedly severer in *age1* than in *age2*. In SSIIa‐NIL, short chains (DP 6–12) and intermediate chains (DP 13–25) were decreased and increased, respectively, as reported previously (Umemoto *et al*., 2004). A combination of *age* and *SSIIa* ^*indica*^ alleles further increased the severity of alteration of CLD of amylopectin. The detailed effects of each allele on the profiles of CLD in the combination lines are considered in the Discussion section.

### *In vitro* digestion profile of starch by α‐amylase

**Figure 8:**
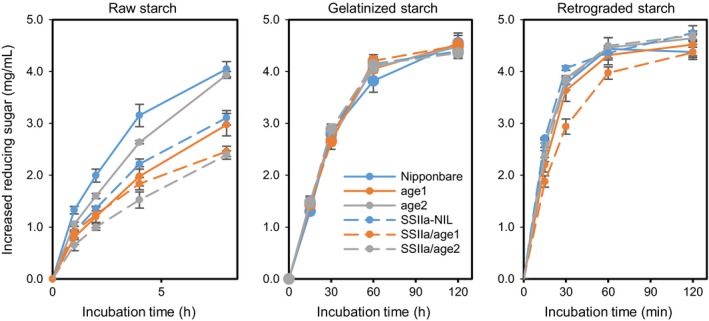
α‐amylase digestion properties of purified starch. Raw, gelatinized or retrograded starches were treated with α‐amylase for indicated time. The amount of reducing sugar produced by the reaction was determined. Values are the means ± SD of three replicates.

To evaluate the digestive property of purified starches, raw, gelatinized and retrograded starches were treated with α‐amylase and generated reducing sugars were determined. Raw starches of *age1*,* age2* and SSIIa‐NIL were digested slower than those of Nipponbare (Figure 8). The digestibility of starches in the double allele lines was further slowed down. Interestingly, although *age2* starch was digested faster than *age1* starch, SSIIa/*age2* showed slower digestibility than SSIIa/*age1*. When starches were gelatinized, the digestive property of the starches of all mutants was comparable to that of Nipponbare (Figure 8). When gelatinized starches were retrograded, only SSIIa/*age1* starch exhibited slower digestibility (Figure 8).

### Eating quality of the cooked rice of the mutant lines

**Figure 9:**
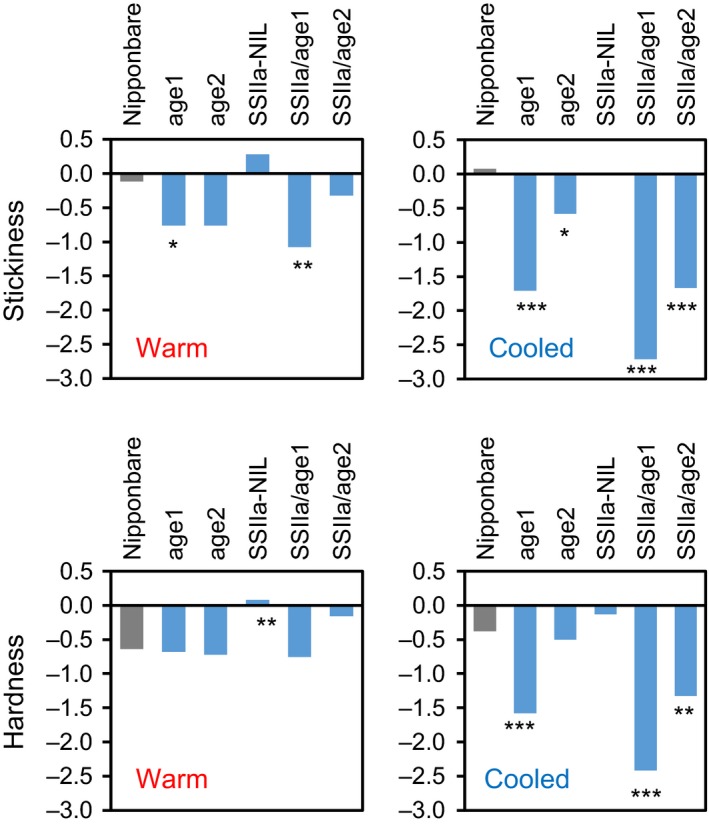
Stickiness and hardness of the cooked rice of the mutant lines as measured by a sensory evaluation test. Each sample was evaluated by 24‐trained panellists under two conditions, in which cooked rice was cooled for 10 m (warm) or 2 h (cooled) at room temperature. For hardness, lower scores are harder texture. Asterisks denote significant differences from Nipponbare (Student's *t* test, *P <* *0.05, **P <* *0.01, ***P <* *0.001).

The eating quality of cooked rice of the mutant lines was evaluated by a sensory evaluation test. A series of the mutants demonstrated stepwise changes of scores related to rice textures including stickiness and hardness. The result with warm rice (10 m after cooking) showed a significant reduction of stickiness in SSIIa/*age1*, leading to the loss of its overall eating quality, as our sensory test grades sticky rice as superior (Figure 9, Table S2). However, the other lines presented various degrees of stickiness and hardness. When the cooked rice was cooled down for 2 h, the variations in stickiness and hardness were more pronounced (Figure 9, Table S2). These parameters of *age1*, SSIIa/*age1* and SSIIa/*age2* were significantly decreased. Especially, SSIIa/*age1* exhibited the least sticky and hard texture. Although SSIIa‐NIL rice exhibited a hard and less sticky texture after prolonged cooling at a lower temperature (e.g. 5 °C for 16 h) (Umemoto *et al*., 2008), it was evaluated as a slightly higher eating quality rice compared to Nipponbare in both tested conditions. However, a combination of alleles with reduced BEIIb activity and *SSIIa* ^*indica*^ allele drastically reduced the scores of most features of eating quality especially in cooled rice. Taken together, a series of materials with a variety of the textures of cooked rice were produced.

## Discussion

### Advantages of application of MutMapPlus

In this study, we isolated *age* mutant lines with moderate changes of grain starch properties and identified them as novel mutants of *BEIIb* by a combination of conventional mapping and MutMapPlus. Before application of MutMapPlus, we tried to identify the causal mutation of *age1* by MutMap. However, the Nipponbare mutant population, from which we found *age* mutants, was generated two decades ago, and the original parental plant subjected to the mutagenesis was not available. Furthermore, as the Nipponbare individuals, which were used for the crossing to establish the F2 progenies were not preserved, we sequenced different individuals to build the reference sequence for the mapping analysis. Under these situations, a trial of MutMap analysis gave multiple candidate regions, where SNP indexes approached 1, throughout the genome due to background SNPs among Nipponbare individuals used for mutagenesis, crossing and reference sequencing, and failed to pick the *age1* causal mutation (Figure S5). So, we employed the MutMapPlus procedure, where the sequences of F2 siblings showing M and WT phenotypes were compared. Although MutMapPlus was originally intended to analyse mutants that are not amenable for crossing, such as sterility, we applied the procedure for *age1* mutant without the available original parental line and successfully narrowed down the candidate SNPs in a single region of the genome. Our result indicated an effective utilization of the MutMapPlus procedure for the mapping of mutants without an available original parental line.

Although we tried to identify the causal mutation in *age2* by MutMapPlus, the analysis alone hardly detected the *Tos17* insertion in *BEIIb* gene because it is difficult to find an indel of a large DNA fragment by this method. However, we could find gaps in every short read aligned to the *Tos17* insertion site when we paid attention to the genomic region around *BEIIb* gene. This fact suggests that, as we conducted in this study, a combination of MutMapPlus with other genetic analyses makes it easy to identify causal mutations including indels.

As BEIIb is well‐studied and characterized for affecting starch properties among the genes in the genomic region identified by rough mapping, it is speculated that if we did not employ MutMapPlus to our analysis, we could focus on *BEIIb* gene and finally identify M723K mutation as the causal mutation corresponding to *age1* phenotype. Application of MutMapPlus, however, markedly shortened the period for identification of the causal mutation and provided solid information about other candidate SNPs around the genomic region. In case of the mapping of agronomic traits with poor information under the situation that the exact reference genome sequence of the original mutagenized plant is not available, MutMapPlus analysis should fully exert its advantages for identification of causal genes/mutations governing the traits. In addition, identification of the causal mutation directly linked with establishment of the DNA marker, which never segregates with the phenotype, to detect the useful allele.

### Biochemical characteristics of BEIIb produced by *age* alleles

Branching enzyme proteins consist of three common characteristic domains, carbohydrate‐binding module 48, catalytic α‐amylase domain and α‐amylase C‐terminal domain (Pfister and Zeeman, 2016). Deletion analysis using chimeric protein of maize BEIIb and BEI revealed that the C‐terminal portion of the C‐terminal domain, which corresponds to a region between Leu‐747 and Glu‐825 of full‐length rice BEIIb, is required for enzyme activity (Hong and Preiss, 2000). In *age2*, insertion of *Tos17* located in a position corresponding to the intermediate region of the C‐terminal domain of *BEIIb* gene would result in the production of inactive BEIIb protein lacking the C‐terminal portion of the C‐terminal domain. This is consistent with the result in which BE activity corresponding to the truncated BEIIb protein was not detected (Figure 2, Figure S6).

Met‐723 residue in rice BEIIb is conserved among BEIIa and BEIIb in maize, wheat and rice and is located within the C‐terminal domain but not in the region required for the enzyme activity described above (Hong and Preiss, 2000). M723K mutation abolished enzyme activity of BEIIb and affected starch properties without a marked decrease in BEIIb protein (Figures 2 and 7, Table 2). However, the degree of the effects on grain quality and amylopectin structure in *age1* was significantly weaker than those in EM10 (Figure S7). One possible explanation of the difference between *age1* and EM10 is the presence of inactive BEIIb protein in *age1*. Starch biosynthetic enzymes in endosperm form large protein complexes (Ahmed *et al*., 2015; Crofts *et al*., 2015; Hennen‐Bierwagen *et al*., 2008; Liu *et al*., 2009, 2012; Tetlow *et al*., 2004, 2008). In maize, *ae1.1,* lacking BEIIb protein, and *ae1.2*, expressing inactive BEIIb, alleles exhibited a different organization and assembly of protein complexes of starch biosynthetic enzymes (Liu *et al*., 2009, 2012). This implies that deficiency of a specific isozyme involved in starch synthesis is compensated by other isozymes through modification of assembly of the protein complexes (Abe *et al*., 2014; Fujita *et al*., 2011; Li and Gilbert, 2016; Tetlow and Emes, 2014). We detected an additional slower migration band only in *age1* extract by IB analysis after native‐PAGE (Figure S6). The protein complex corresponding to the band is considered to be exclusively organized by M723K‐mutated BEIIb. In addition, composition of starch‐bound proteins was altered in *age1* (Figure S1) as observed in maize inactive BEIIb mutant *ae1.2* (Liu *et al*., 2012). M723K mutation slightly up‐regulated starch‐binding ability of BEIIb (Figure S1). These results indicate that physical protein–protein interactions between starch biosynthetic enzymes and their starch‐binding states are rearranged in *age1* by the expression of M723K‐mutated BEIIb. These alterations may lead to partial compensation of BEIIb function in *age1*. Further detailed researches concerning the contribution of starch‐bound and unbound respective enzymes to the altered starch properties are necessary.

Compared to single *age* lines, SSIIa/*age* double allele lines showed a severe effect on starch properties (Figures 1a and 8, Table 2). The decline in BEIIb activity by *age* alleles restrains generation of new short branches by cleaving longer chains, and active *SSIIa* ^*indica*^ allele increases longer chains by elongation of short branches during amylopectin synthesis, resulting in production of amylopectin composed of less‐branched longer glucan chains in crystalline lamellae (Figure 7). Longer glucan chains in amylopectin also increase apparent amylose content (Table 2). As the proportion of short chains (DP < 13) of amylopectin is correlated with the digestibility and gelatinization temperature of starch positively and negatively, respectively (Tanaka *et al*., 2004; Zhang *et al*., 2008), profound effects on starch properties in the double allele lines are explained by alteration of CLD of amylopectin.

We analysed the contribution of each allele in the double allele lines to CLD of amylopectin (Figure S8). The contribution of *age* alleles in the single and double allele lines to the distribution of intermediate to long glucan chains (DP > 14) was almost the same, whereas those to short chains (DP 6–12) were different. A similar trend was observed in the effect of *SSIIa* ^*indica*^ allele. The effect of *age1* and *SSIIa* ^*indica*^ on starch properties was severely attenuated in SSIIa^*indica*^/*age1* compared to that of *age2* and *SSIIa* ^*indica*^ in SSIIa^*indica*^/*age2* (Figure S8), resulting in a similar degree of effects of *age1* and *age2* in the double allele lines on starch properties (Figure 8, Table 2). We hypothesized that BEIIb and SSIIa act independently for synthesizing longer chains, whereas they have functional interaction, which is probably originated from physical interactions of these enzymes (Crofts *et al*., 2015), to some extent, for making short chains. According to the hypothesis, CLD of amylopectin in *ss3a ss4b* double mutant showed a different pattern from that in *ss3a* and *ss4b* single mutants (Toyosawa *et al*., 2016). In contrast, two double mutants, *ss1 be1* and *ss1 be2b*, displayed additive patterns of their respective single mutants in CLD of amylopectin (Abe *et al*., 2014). Accumulation of understandings from these approaches should contribute to interpret physiological functions of the organization of a specific protein complex by starch biosynthesis‐related proteins during starch biosynthesis and uncover the missing links in the mechanisms of starch biosynthesis in future studies.

### Availability and advantages of *age* alleles for breeding

BEIIb‐deficient mutants have been reported to produce rice grain with a chalky appearance, which reflects the morphology of starch granules (Kubo *et al*., 2010). Rice grains with chalky appearance reduce the yield of head rice after polishing and lower their market value (Fitzgerald *et al*., 2009). In contrast, production of almost normal grains in *age1* and *age2* may be involved in moderate alteration of their starch properties (Figure 1b). Although occasional production of white core grains in *age1* and *age2* was observed regardless of their growth conditions in the paddy field, greenhouse and plant incubator, the ratios of white core grain and severity of chalkiness in *age* grains are significantly lower than those in EM10 (Figure S7b). In addition, the effects of *age* alleles on grain size and weight were weak (Table 2). The less negative effect of *age* alleles on grain appearance is not presumed to reduce the market value of the rice, being a great advantage in the utilization of *age* alleles for breeding.

In conclusion, comprehensive assessment of biochemical and food chemical characteristics demonstrated that *age* alleles produce starch with moderately changed properties and are capable of utilization for practical breeding. Development of DNA markers linked with the alleles can accelerate utilization of these alleles. Recently, consumers’ preferences became diversified owing to the variety of food cultures. Markets require a wide range of cooked rice textures, not only traditionally preferred soft rice but also less sticky rice. Rice breeders can select one or several lines produced in this study for breeding depending on the objective of breeding. We hope that our work facilitates rice breeding towards new cultivars with novel features of cooked rice.

## Experimental procedures

Plant growth, screening of *age* mutants, mapping of mutant genes, plasmid construction, generation of transgenic plants, measurements of starch properties, RT‐PCR and protein analyses were performed according to previous reports with slight modification (Asai *et al*., 2014; Fujita *et al*., 1999; Hirochika, 2001; Kosugi *et al*., 2013; Kuroda *et al*., 2010; Li and Durbin, 2009; Li *et al*., 2009; Miyao *et al*., 2003; Nakagawa *et al*., 2007; Nelson, 1944; Toki, 1997; Yamakawa *et al*., 2007). Details of the Supplementary Experimental Procedures were provided in the Appendix S1. Primers used in this study and antigens for raising specific polyclonal antibodies are listed in Tables S3 and S4, respectively.

## Supporting information


**Figure S1** Distribution of starch biosynthetic enzymes in developing endosperm. Immunoblot (IB) analysis of soluble protein (SP) and proteins loosely (LBP) or tightly (TBP) bound to starch granule in developing seeds. Protein fractions were prepared as reported by Asai *et al*. (2014). Antibodies used were indicated below the images. Positions of molecular markers were shown on the left of the images.

**Figure S2** SNP index and Δ(SNP index) plots of rice chromosomes generated by MutMapPlus analysis of *age1*. SNP index and Δ(SNP index) plots were generated by MutMapPlus analysis of bulked F2 mutant progeny obtained by a crossing between *age1* and Nipponbare. (a) SNP index of the mutant (M) bulk. (b) SNP index of the wild‐type (WT) bulk. (c) Δ(SNP index) calculated by subtraction of the index of WT bulk from that of M bulk. Red lines indicate average value of SNP index or Δ(SNP index) obtained by the sliding window analysis of 4 Mb intervals with 50 kb increment. Candidate region confined by the Fisher's *P* value of <0.05 is indicated with a red box.

**Figure S3** Investigation of urea concentration for gelatinization of starch of vector control lines of Nipponbare and *age1*. Halved rice grains were incubated with various concentrations of urea solution overnight and gelatinization of starch was evaluated by iodine staining. Transgenic plants were grown in a greenhouse. Temperatures after flowering were set at 27 °C/22 °C during 15 h light/9 h dark periods, respectively.

**Figure S4** Investigation of urea concentration for gelatinization of endosperm starch of F1 seed in allelism test. Endosperm powder of F1 seeds (2.5 mg) was incubated with various concentrations of urea solution (150 μL) and gelatinization of starch was evaluated by the volumes of sediments. Grains used for this experiment were ripened in a warm and closed room in summer (see Experimental procedures).

**Figure S5** Trial of mapping *age1* mutation by MutMap analysis. SNP index plot was generated by MutMap analysis of bulked F2 mutant progeny obtained by a crossing between *age1* and Nipponbare. Red lines indicate average value of SNP index obtained by the sliding window analysis of 4 Mb interval with 50 kb increment. Green lines represent the 95% statistical confidence limit.

**Figure S6** BE staining and immunoblot analyses after native‐PAGE. Ten micrograms of soluble endosperm extract was separated by native‐PAGE. After electrophoresis, the gels were subjected to BE activity staining or immunoblot analyses with anti‐BEIIb antibody. The slight delay in the migration of the immunoreactive band of *age1* occurred probably due to the change in electrical charge by the M723K substitution. An additional band observed in *age1* was indicated by a red arrowhead.

**Figure S7** Comparison of EM10 and *age1*. (a) The distribution of the length of amylopectin side chains was examined by HPAEC‐PAD, and then the difference in the profiles between EM10 and Kinmaze or *age1* and Nipponbare was calculated by subtracting the ratio of a chain of given length of the sample with that of the mean value of Kinmaze or Nipponbare, respectively. Values are the means of three biological replicates. (b) Grain appearance of *age1* and EM10. Images by reflected light (left panel) and transmitted light (right panel) are shown. Grains used in this experiment were ripened in a plant incubator.

**Figure S8** Comparison of chain length distribution between single and double allele lines. The difference in the chain length profiles of amylopectin between the double allele lines and their parental single allele lines was shown. The values were calculated by subtracting the ratio of a chain of given length of the double allele line with that of the single allele line shown in Figure 7.

**Table S1** SNPs identified in *age2* by BWA mapping and Coval extraction between 10 Mb and 3′ end of chromosome 2.

**Table S2** Eating quality of the cooked rice of the mutant lines as measured by a sensory evaluation test.

**Table S3** Primers used in this study.

**Table S4** Peptide sequences of antigens for raising polyclonal antibodies.

**Appendix S1** Experimental procedures.
