# Supplementary material for: MutMapPlus identified novel mutant alleles of a rice starch branching enzyme IIb gene for fine‐tuning of cooked rice texture
Source: Plant Biotechnol J. 2017 Jun 14;16(1):111–23. doi: 10.1111/pbi.12753 (PMC5785365; doi:10.1111/pbi.12753)
Supplement: Supplementary file 1 — Figure S1 Distribution of starch biosynthetic enzymes in developing endosperm. Immunoblot (IB) analysis of soluble protein (SP) and proteins loosely (LBP) or tightly (TBP) bound to starch granule in developing seeds. Protein fractions were prepared as reported by Asai et al. (2014). Antibodies used were indicated below the images. Positions of molecular markers were shown on the left of the images. [file PBI-16-111-s010.pdf]

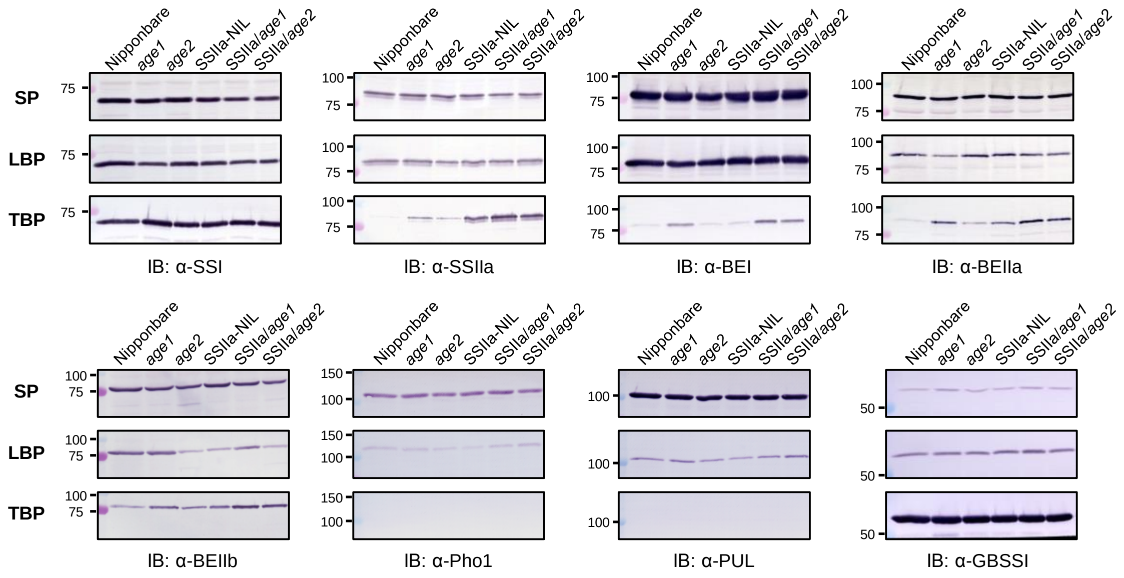

Figure S1. Distribution of starch biosynthetic enzymes in developing endosperm.

Immunoblot (IB) analysis of soluble protein (SP) and proteins loosely (LBP) or tightly (TBP) bound to starch granule in developing seeds. Protein fractions were prepared as reported by Asai *et al*. (2014). Antibodies used were indicated below the images. Positions of molecular markers were shown on the left of the images.
