# Supplementary material for: MutMapPlus identified novel mutant alleles of a rice starch branching enzyme IIb gene for fine‐tuning of cooked rice texture
Source: Plant Biotechnol J. 2017 Jun 14;16(1):111–23. doi: 10.1111/pbi.12753 (PMC5785365; doi:10.1111/pbi.12753)
Supplement: Supplementary file 2 — Figure S2 SNP index and Δ(SNP index) plots of rice chromosomes generated by MutMapPlus analysis of age1. SNP index and Δ(SNP index) plots were generated by MutMapPlus analysis of bulked F2 mutant progeny obtained by a crossing between age1 and Nipponbare. (a) SNP index of the mutant (M) bulk. (b) SNP index of the wild‐type (WT) bulk. (c) Δ(SNP index) calculated by subtraction of the index of WT bulk from that of M bulk. Red lines indicate average value of SNP index or Δ(SNP index) obtained by the sliding window analysis of 4 Mb intervals with 50 kb increment. Candidate region confined by the Fisher's P value of <0.05 is indicated with a red box. [file PBI-16-111-s009.pdf]

(a)

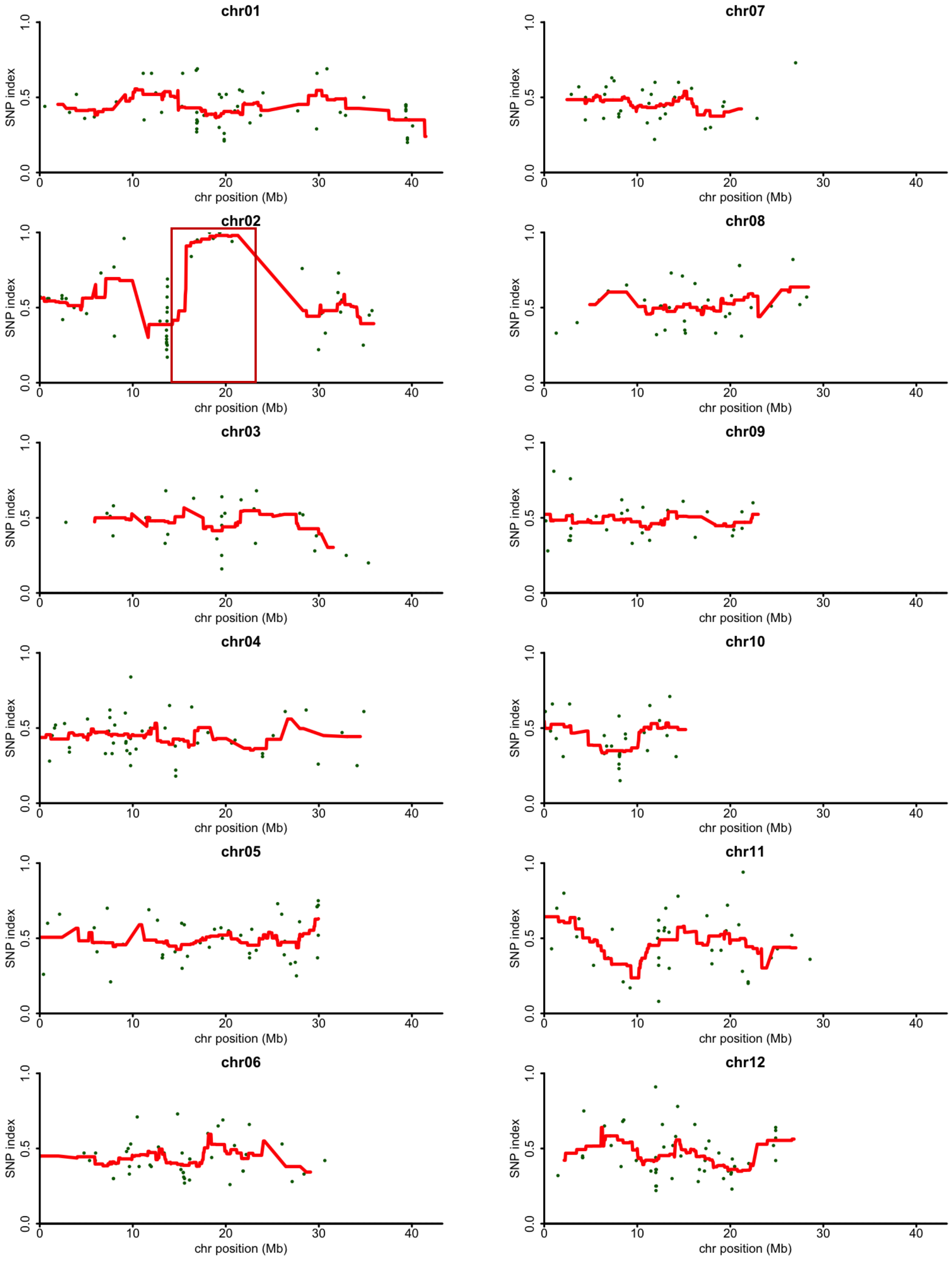

Figure S2. SNP index and Δ(SNP index) plots of rice chromosomes generated by MutMapPlus analysis of *age1*.

SNP index and Δ(SNP index) plots were generated by MutMapPlus analysis of bulked F2 mutant progeny obtained by a crossing between *age1* and Nipponbare. (a) SNP index of the mutant (M) bulk. (b) SNP index of the wild-type (WT) bulk. (c) Δ(SNP index) calculated by subtraction of the index of WT bulk from that of M bulk. Red lines indicate average value of SNP index or Δ(SNP index) obtained by the sliding window analysis of 4 Mb intervals with 50 kb increment. Candidate region confined by the Fisher's *P* value of <0.05 is indicated with a red box.

(b)

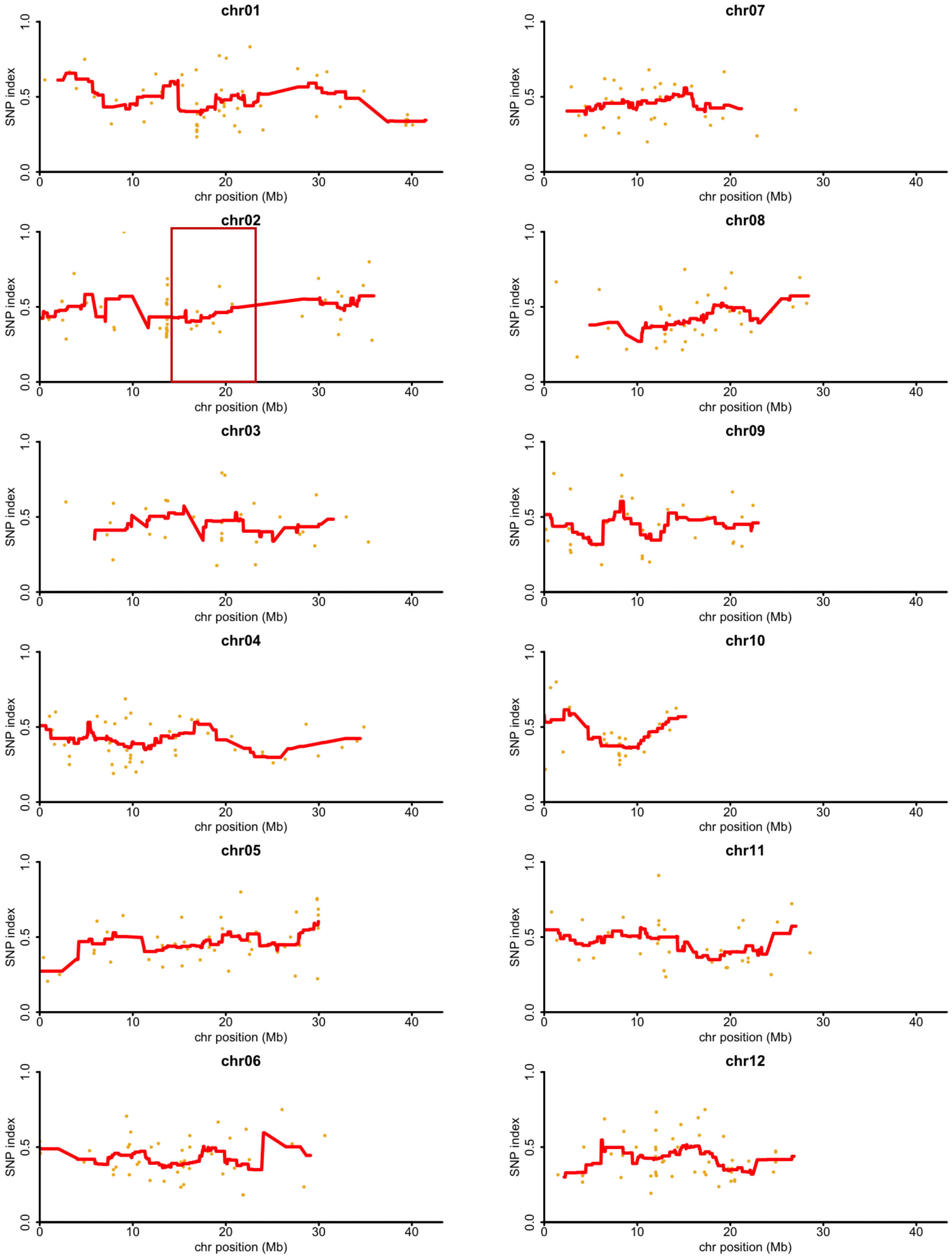

Figure S2. (Continued)

(c)

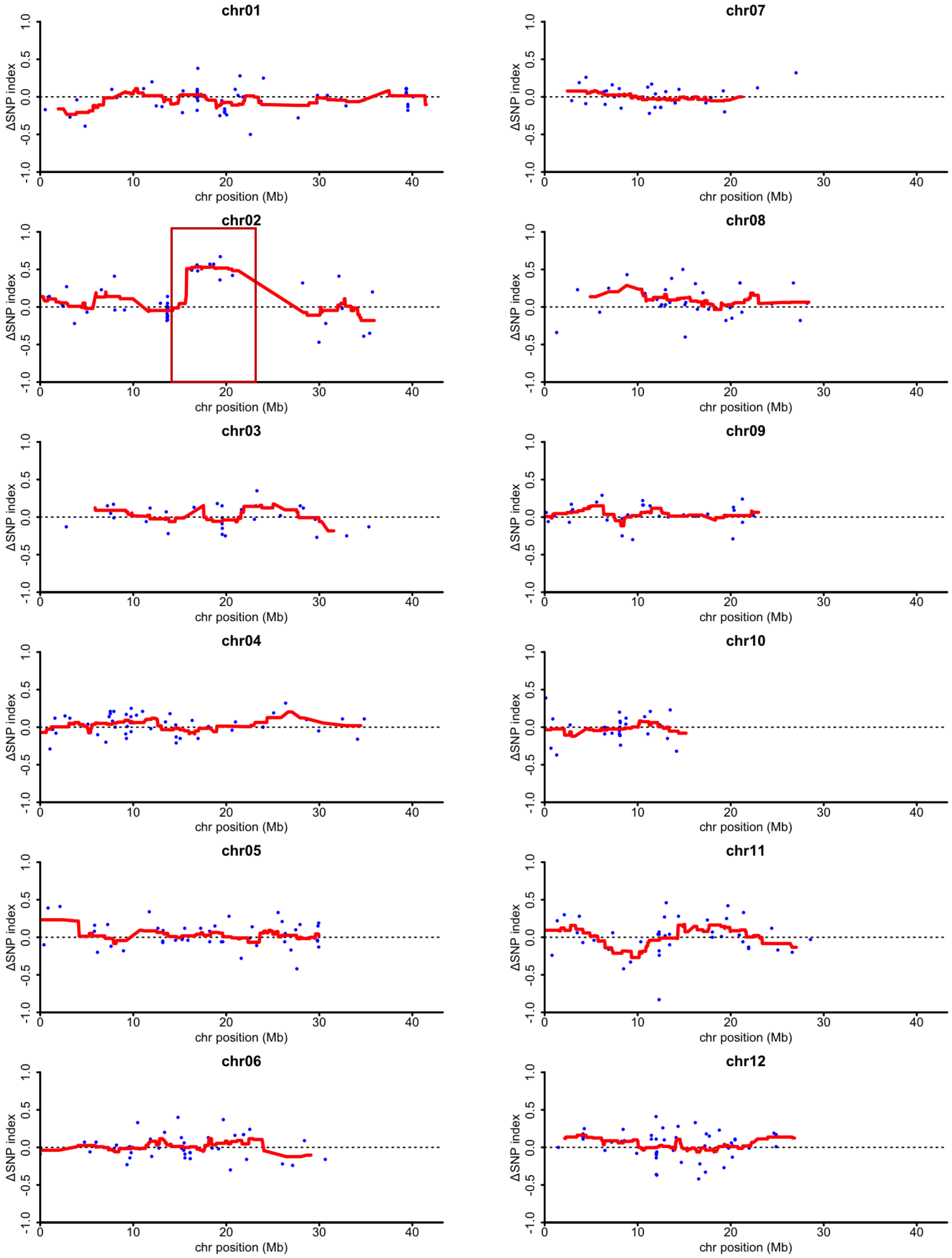

Figure S2. (Continued)
