# Supplementary material for: MutMapPlus identified novel mutant alleles of a rice starch branching enzyme IIb gene for fine‐tuning of cooked rice texture
Source: Plant Biotechnol J. 2017 Jun 14;16(1):111–23. doi: 10.1111/pbi.12753 (PMC5785365; doi:10.1111/pbi.12753)
Supplement: Supplementary file 3 — Figure S3 Investigation of urea concentration for gelatinization of starch of vector control lines of Nipponbare and age1. Halved rice grains were incubated with various concentrations of urea solution overnight and gelatinization of starch was evaluated by iodine staining. Transgenic plants were grown in a greenhouse. Temperatures after flowering were set at 27 °C/22 °C during 15 h light/9 h dark periods, respectively. [file PBI-16-111-s008.pdf]

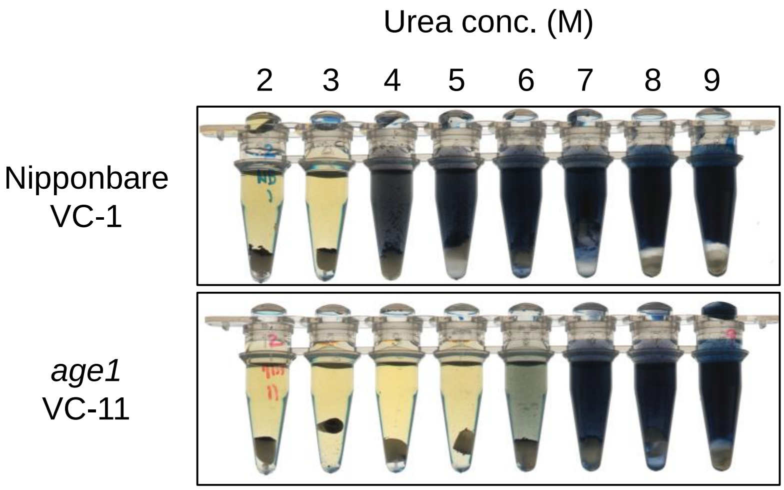

Figure S3. Investigation of urea concentration for gelatinization of starch of vector control lines of Nipponbare and *age1*.
Halved rice grains were incubated with various concentrations of urea solution overnight and gelatinization of starch was evaluated by iodine staining. Transgenic plants were grown in a greenhouse. Temperatures after flowering were set at 27° C/22° C during 15 h light/9 h dark periods, respectively.
