# Supplementary material for: MutMapPlus identified novel mutant alleles of a rice starch branching enzyme IIb gene for fine‐tuning of cooked rice texture
Source: Plant Biotechnol J. 2017 Jun 14;16(1):111–23. doi: 10.1111/pbi.12753 (PMC5785365; doi:10.1111/pbi.12753)
Supplement: Supplementary file 4 — Figure S4 Investigation of urea concentration for gelatinization of endosperm starch of F1 seed in allelism test. Endosperm powder of F1 seeds (2.5 mg) was incubated with various concentrations of urea solution (150 μL) and gelatinization of starch was evaluated by the volumes of sediments. Grains used for this experiment were ripened in a warm and closed room in summer (see Experimental procedures). [file PBI-16-111-s012.pdf]

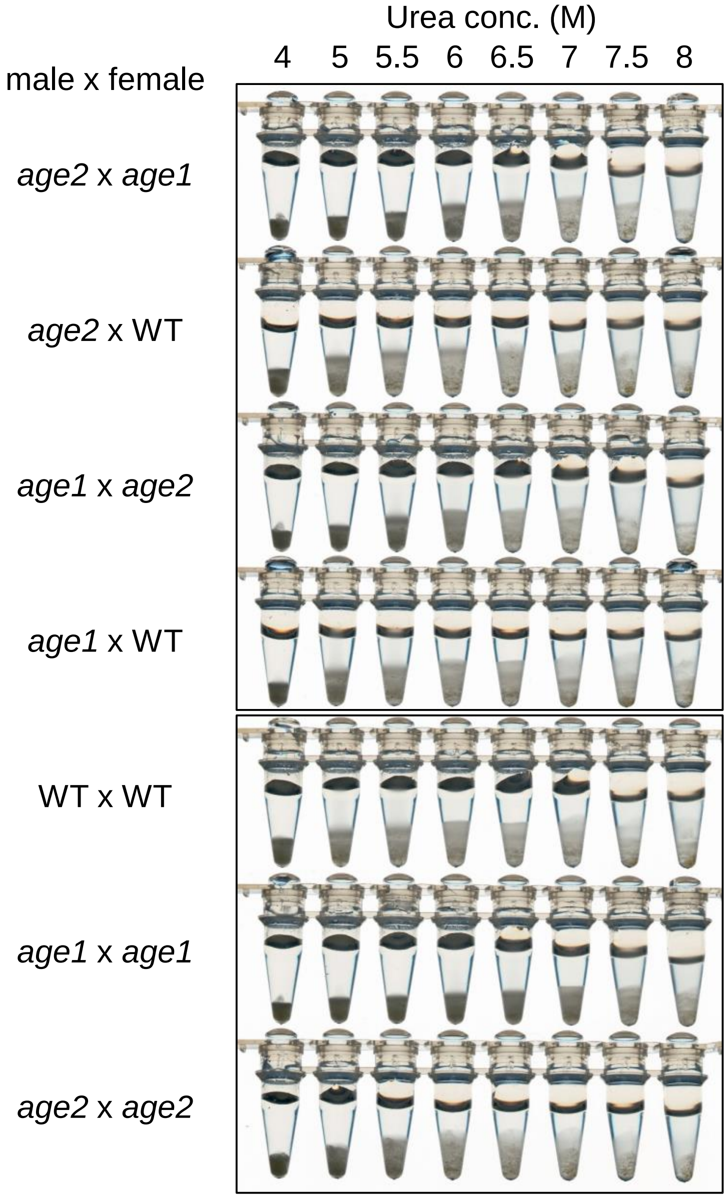

Figure S4. Investigation of urea concentration for gelatinization of endosperm starch of F1 seed in allelism test.

Endosperm powder of F1 seeds (2.5 mg) was incubated with various concentrations of urea solution (150 μl) and gelatinization of starch was evaluated by the volumes of sediments. Grains used for this experiment were ripened in a warm and closed room in summer (see Experimental procedures).
