# Supplementary material for: MutMapPlus identified novel mutant alleles of a rice starch branching enzyme IIb gene for fine‐tuning of cooked rice texture
Source: Plant Biotechnol J. 2017 Jun 14;16(1):111–23. doi: 10.1111/pbi.12753 (PMC5785365; doi:10.1111/pbi.12753)
Supplement: Supplementary file 5 — Figure S5 Trial of mapping age1 mutation by MutMap analysis. SNP index plot was generated by MutMap analysis of bulked F2 mutant progeny obtained by a crossing between age1 and Nipponbare. Red lines indicate average value of SNP index obtained by the sliding window analysis of 4 Mb interval with 50 kb increment. Green lines represent the 95% statistical confidence limit. [file PBI-16-111-s001.pdf]

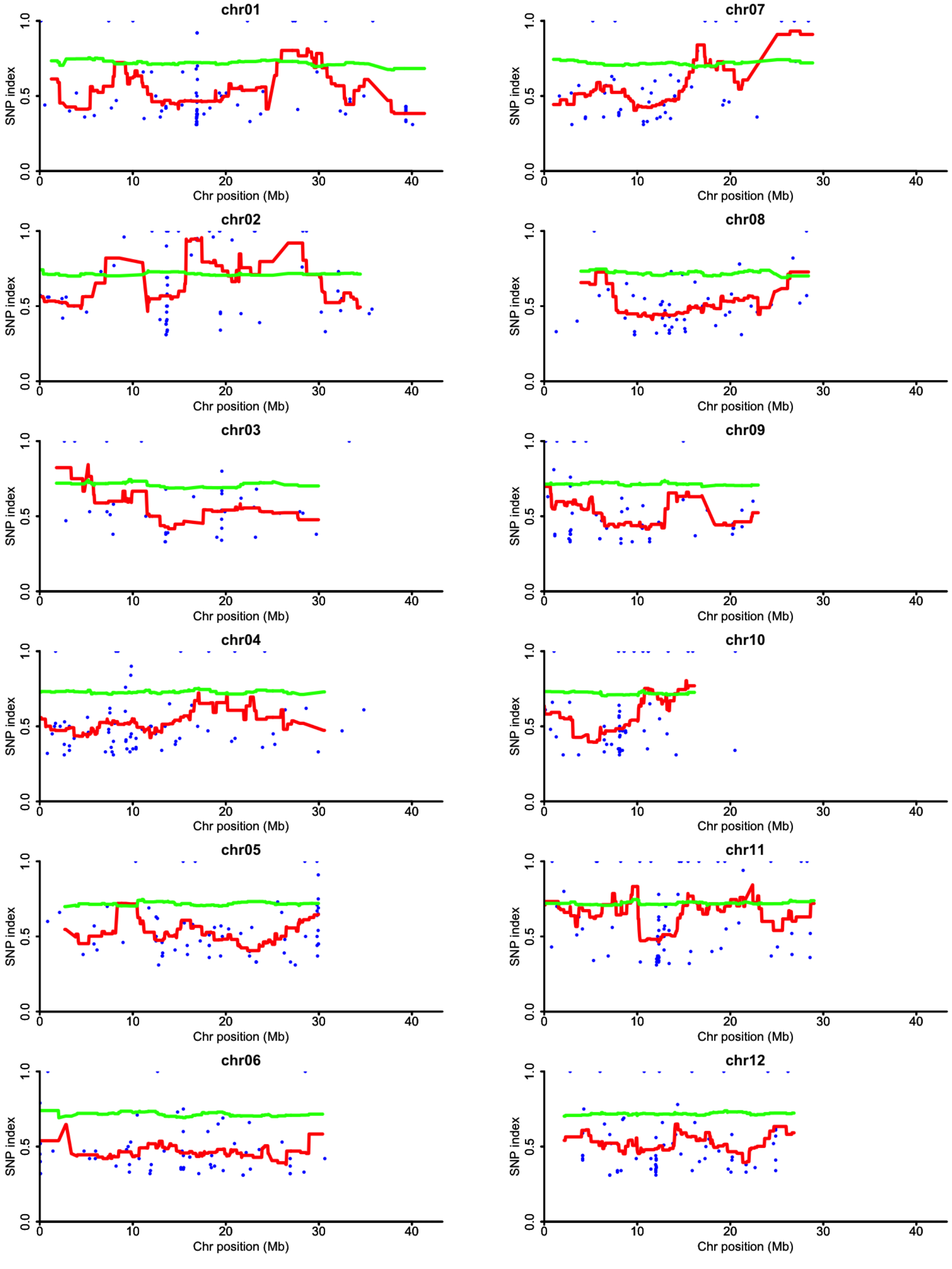

Figure S5. Trial of mapping *age1* mutation by MutMap analysis.
SNP index plot was generated by MutMap analysis of bulked F2 mutant progeny obtained by a crossing between *age1* and Nipponbare. Red lines indicate average value of SNP index obtained by the sliding window analysis of 4 Mb interval with 50 kb increment. Green lines represent the 95% statistical confidence limit.
