# Supplementary material for: MutMapPlus identified novel mutant alleles of a rice starch branching enzyme IIb gene for fine‐tuning of cooked rice texture
Source: Plant Biotechnol J. 2017 Jun 14;16(1):111–23. doi: 10.1111/pbi.12753 (PMC5785365; doi:10.1111/pbi.12753)
Supplement: Supplementary file 6 — Figure S6 BE staining and immunoblot analyses after native‐PAGE. Ten micrograms of soluble endosperm extract was separated by native‐PAGE. After electrophoresis, the gels were subjected to BE activity staining or immunoblot analyses with anti‐BEIIb antibody. The slight delay in the migration of the immunoreactive band of age1 occurred probably due to the change in electrical charge by the M723K substitution. An additional band observed in age1 was indicated by a red arrowhead. [file PBI-16-111-s002.pdf]

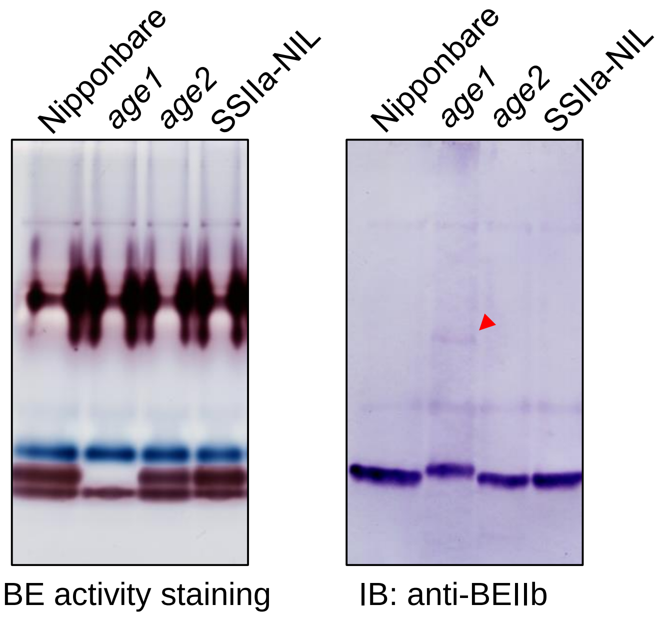

Figure S6. BE staining and immunoblot analyses after native-PAGE.
Ten micrograms of soluble endosperm extract was separated by native-PAGE. After electrophoresis, the gels were subjected to BE activity staining or immunoblot analyses with anti-BEIIb antibody. The slight delay in the migration of the immunoreactive band of *age1* occurred probably due to the change in electrical charge by the M723K substitution. An additional band observed in *age1* was indicated by a red arrowhead.
