# Supplementary material for: MutMapPlus identified novel mutant alleles of a rice starch branching enzyme IIb gene for fine‐tuning of cooked rice texture
Source: Plant Biotechnol J. 2017 Jun 14;16(1):111–23. doi: 10.1111/pbi.12753 (PMC5785365; doi:10.1111/pbi.12753)
Supplement: Supplementary file 7 — Figure S7 Comparison of EM10 and age1. (a) The distribution of the length of amylopectin side chains was examined by HPAEC‐PAD, and then the difference in the profiles between EM10 and Kinmaze or age1 and Nipponbare was calculated by subtracting the ratio of a chain of given length of the sample with that of the mean value of Kinmaze or Nipponbare, respectively. Values are the means of three biological replicates. (b) Grain appearance of age1 and EM10. Images by reflected light (left panel) and transmitted light (right panel) are shown. Grains used in this experiment were ripened in a plant incubator. [file PBI-16-111-s003.pdf]

(a)

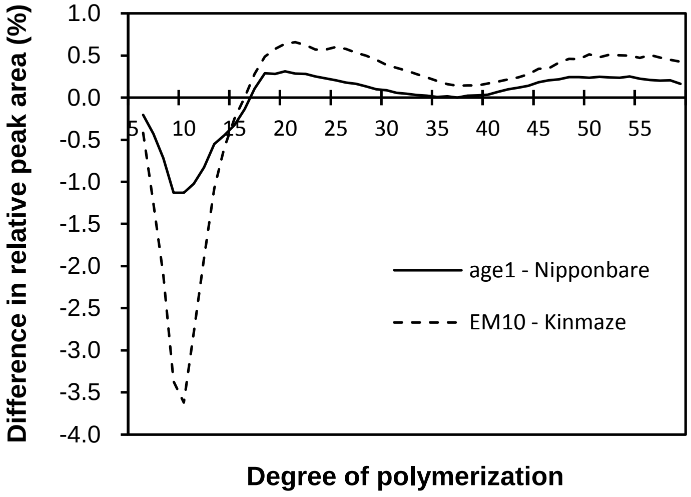

(b)

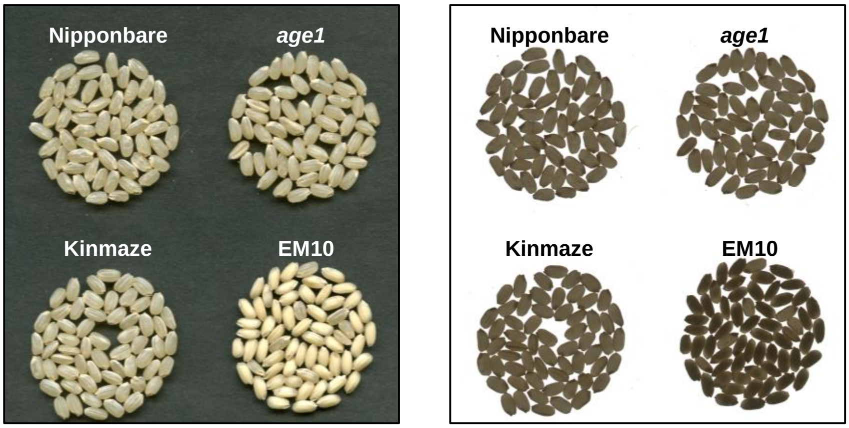

Figure S7. Comparison of EM10 and *age1*.
(a) The distribution of the length of amylopectin side chains was examined by HPAEC-PAD, and then the difference in the profiles between EM10 and Kinmaze or *age1* and Nipponbare was calculated by subtracting the ratio of a chain of given length of the sample with that of the mean value of Kinmaze or Nipponbare, respectively. Values are the means of three biological replicates. (b) Grain appearance of *age1* and EM10. Images by reflected light (left panel) and transmitted light (right panel) are shown. Grains used in this experiment were ripened in a plant incubator.
