# Supplementary material for: MutMapPlus identified novel mutant alleles of a rice starch branching enzyme IIb gene for fine‐tuning of cooked rice texture
Source: Plant Biotechnol J. 2017 Jun 14;16(1):111–23. doi: 10.1111/pbi.12753 (PMC5785365; doi:10.1111/pbi.12753)
Supplement: Supplementary file 8 — Figure S8 Comparison of chain length distribution between single and double allele lines. The difference in the chain length profiles of amylopectin between the double allele lines and their parental single allele lines was shown. The values were calculated by subtracting the ratio of a chain of given length of the double allele line with that of the single allele line shown in Figure 7. [file PBI-16-111-s004.pdf]

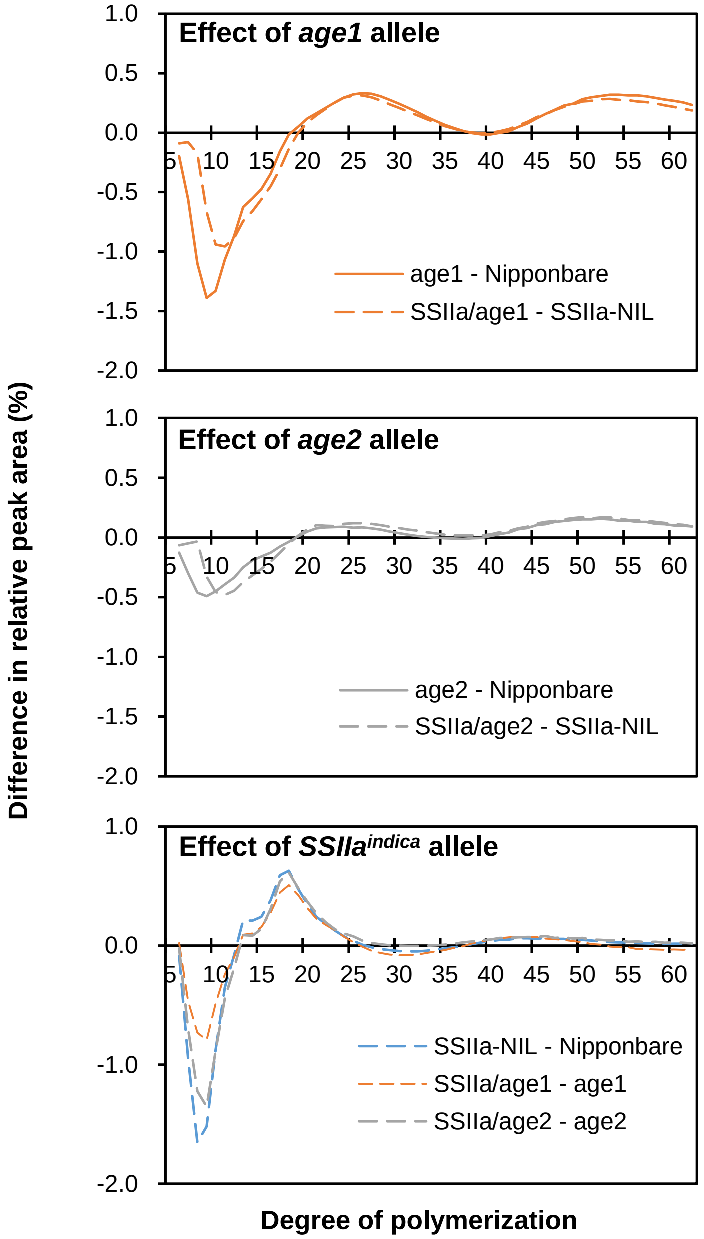

Figure S8. Comparison of chain length distribution between single and double allele lines.

The difference in the chain length profiles of amylopectin between the double allele lines and their parental single allele lines was shown. The values were calculated by subtracting the ratio of a chain of given length of the double allele line with that of the single allele line shown in Figure 7.
