# Supplementary material for: MutMapPlus identified novel mutant alleles of a rice starch branching enzyme IIb gene for fine‐tuning of cooked rice texture
Source: Plant Biotechnol J. 2017 Jun 14;16(1):111–23. doi: 10.1111/pbi.12753 (PMC5785365; doi:10.1111/pbi.12753)
Supplement: Supplementary file 13 — Appendix S1 Experimental procedures. [file PBI-16-111-s011.docx]

**SUPPORTING INFORMATION**

**Experimental procedures**

**Plant materials and growth conditions**

*Tos17* insertion mutant library of a *japonica* cultivar Nipponbare was provided from Rice Genome Resource Center, National Institute of Agrobiological Sciences, Japan (Miyao *et al*., 2003). Rice plants were grown in the paddy field and greenhouse of Hokuriku Research Station, Central Region Agricultural Research Center, National Agriculture and Food Research Organization (Joetsu, Japan). Plant incubators (model NC350H or LPH-410SPC; NIPPON MEDICAL & CHEMICAL INSTRUMENT, Osaka, Japan) were also used as described previously (Yamakawa *et al.*, 2007). Mean air temperatures in the latter half (15th to 31st) of August, corresponding to the grain filling period for Nipponbare and its derived mutants were 25.0°C, 28.4°C, 24.4°C, and 26.5°C in 2004, 2012, 2015, and 2016, respectively.

For allelism test, crossings were performed during the middle of August. Crossed rice plants were ripened indoors until maturation, without temperature control.

**Screening of *age* mutants by urea digestibility test**

Mutants displaying altered gelatinization of grain starch were obtained from the Nipponbare *Tos17* insertion mutant library, in which a prolonged tissue culture induced various types of spontaneous mutation including insertion of endogenous retrotransposons and nucleotide substitution (Hirochika, 2001). Approximately 3,300 independent lines of mutant seeds, which had been harvested in 2004, were screened for altered gelatinization. Sixteen brown rice seeds per each line were halved by cutting with a surgical knife, and the half of each grain was placed in a well of a 96-well plate. Then, 200 μl of 3.6 M urea solution was added. Following incubation at 24°C for 16 h, the gelatinization of starch was detected by staining disintegrated starch with 20 μl of 0.2% (w/v) I_2_, 1% (w/v) KI, and 0.5% (v/v) acetic acid.

The line number of mutants corresponding to *age1* and *age2* is NE7134 and NE9195 in the library, respectively.

**Measurement of gelatinization properties**

Halved grains not containing embryo were incubated with 200 μl of urea solution at concentrations as indicated at room temperature overnight. Starch gelatinization was evaluated by staining of disintegrated starch by 20 μl of iodine solution containing 0.2% (w/v) I_2_, 1% (w/v) KI, and 0.5% (v/v) acetic acid.

For allelism test, 5 to 8 F1 grains, whose embryo was removed, were ground using Multi-Beads Shocker (Yasui Kikai, Osaka, Japan) and 2.5 mg of the resultant powder was mixed with 150 μl of urea solution at concentrations as indicated. After incubation at room temperature overnight, gelatinization of starch granules was evaluated by the volume of swollen sediment.

**Mapping of *age* causal mutations**

The *age1*and *age2* mutants, whose genetic background is a *japonica* cultivar, Nipponbare, were crossed to another japonica cultivar, Koshihikari, and the resulting F1 plants were self-pollinated, producing F2 seeds. The gelatinization phenotype of halved *age1* and *age2* F2 grains, which did not bear embryo, were evaluated using 6.0 M and 4.5 M urea solutions, respectively, as described above. The grains which were not gelatinized were designated mutant (M) phenotypes, the embryo was allowed to germinate, and they were subjected to genomic DNA extraction by the CTAB method. The extracted DNA samples from 43 and 48 M-type individuals among *age1* and *age2* segregants were applied to the Illumina Golden Gate genotyping assay as described previously (Yamamoto *et al*., 2010) for coarse mapping.

Next, the mutant lines were crossed to SL09, which is a chromosome segment substitution line containing a genome segment of an *indica* cultivar, Kasalath, for that region on the Nipponbare background genome, and the gelatinization phenotype of the derived F2 progenies was evaluated using 5.5 M and 4.5 M urea solutions for the *age1* and *age2* mutation, respectively, as described above. The 71 and 89 individuals exhibiting the M phenotypes were subjected to genotyping using the SSR markers listed in Table S3.

For the MutMapPlus analysis, the *age1* and *age2* mutants were crossed to the parental cultivar, Nipponbare, and the derived F2 progenies were evaluated for the gelatinization phenotype using 5.8 M and 4.5 M urea solutions for the *age1* and *age2* mutation, respectively, as described above, and designated M and wild-type (WT) individuals for those not gelatinized and gelatinized in the urea solution, respectively. Genomic DNA was extracted from the leaves of F2 segregants as well as Nipponbare, which is used as the reference, using a DNeasy Plant Mini Kit (QIAGEN, Chatsworth, CA). By combining an equal amount of DNA from each of 25 F2 individuals showing M and WT phenotypes, the respective bulked DNA samples were prepared. The sequencing library was constructed with TruSeq DNA PCR-Free Sample Preparation Kit (Illumina, San Diego, CA) and subjected to 150-bp paired end sequencing by an Illumina HiSeqX DNA sequencer. The data analysis was performed with the MutMapPlus pipeline, which was downloaded from Iwate Biotechnology Research Center (http://genome-e.ibrc.or.jp/home/bioinformatics-team/mutmap), with modification of removal of the filter for extraction of G to A and C to T base conversions. The yielded short reads derived from M and WT bulked samples were filtered by phred quality score with the default setting and aligned to the Nipponbare reference genome sequence (IRGSP-1.0) using the BWA software (Li and Durbin, 2009), and the alignments were converted to BAM files by SAMtools (Li *et al*., 2009). The SNPs with low quality score were excluded with the Coval filter (Kosugi *et al*., 2013), and SNPs which were detected by the self-alignment of the Nipponbare short reads to the above reference sequence were excluded from the analysis. Then, SNP index and Δ(SNP index) were calculated in cases where the SNPs were detected in both of the M and WT bulks. After extraction of the SNPs that match the following criteria: 1) the SNP index of either bulk was higher than 0.3 and 2) the number of reads at the position was higher than 10 and lower than 100 in both bulks, sliding window analysis was performed with 4 Mb window size and 50 kb increase with the R script included in the pipeline in cases that 3 or more SNPs were detected within the window, and gave the average SNP index and *P* value in Fisher’s exact test for respective SNPs. Then, the candidates for the causal mutation were confined by selecting SNPs exhibiting a *P* value of <0.05. For the MutMap analysis, its pipeline was downloaded from Iwate Biotechnology Research Center, and the above M bulk sequence was used for the analysis with the same modification for the SNP filtration step.

**Plasmid construction and generation of transgenic plants**

For expression of WT *BEIIb*, the genomic fragment of *BEIIb* gene (about 14.5 kb) was amplified from Nipponbare genomic DNA with the primers BEIIb-AscI-F and BEIIb-SalI-R (Table S3). The PCR product was digested with *Asc*I and *Sal*I and cloned into the corresponding restriction enzyme sites of the pZH2B binary vector (Kuroda *et al.*, 2010). For production of M723K mutant of *BEIIb*, two genomic fragments of *BEIIb* gene amplified from *age1* genomic DNA with two sets of primers, BEIIb-AscI-F and M723K-R, and M723K-F and BEIIb-SalI-R (Table S3). These PCR products were used for templates of the second PCR using primers BEIIb-AscI-F and BEIIb-SalI-R. The PCR product was cloned into pZH2B as described above.

For insertion of four times-repeated Myc tag into the C-terminal of BEIIb, two DNA fragments corresponding to the tag and 3’ region of *BEIIb* gene were amplified using pUGW16 (Nakagawa *et al.*, 2007) and Nipponbare genomic DNA as templates with primers ClaI-myc-F and myc-UTR-R, and myc-UTR-F and BEIIb-SalI-R (Table S3), respectively. These PCR products were used for templates of the second PCR with primers ClaI-myc-F and BEIIb-SalI-R to generate Myc-3’UTR fragment. The genomic region of *BEIIb* gene with or without M723K mutation was amplified with primers BEIIb-AscI-F and BEIIb-ClaI-R using binary vectors described above as templates. The PCR product and Myc-3’UTR fragment were digested with *Asc*I and *Cla*I, and *Cla*I and *Sal*I, respectively, and then ligated with pZH2B vector digested with *Asc*I and *Sal*I.

The binary vectors were introduced into *Agrobacterium tumefaciens* strain EHA101 by electroporation. Transformation of Nipponbare and *age1* was performed as described (Toki, 1997).

**RNA extraction and RT-PCR**

Total RNA was extracted from three caryopses of 12 days after flowering (DAF) by RNeasy Plant Mini Kit (QIAGEN). First-strand cDNA was synthesized from 0.5 μg of total RNA with oligo (dT) primer using PrimeScript RT reagent kit (Takara-Bio, Kusatsu, Japan). Quantitative RT-PCR was performed with Thermal Cycler Dice Real Time System TP850 (Takara-Bio) using SYBR Premix Ex Taq (Tli RNaseH Plus) (Takara-Bio) and gene-specific primer sets listed in Table S3. Relative amounts of transcripts were calculated by relative quantification method with standard curve using the *eEF-1α* gene as an internal control. Three biological replicates were included in each experiment.

For detection of splice variants in *age2*, cDNA or total RNA was used as a template. PCR was performed using 0.25 U of PrimeSTAR GXL DNA polymerase (Takara-Bio) and the primers listed in Table S3 with the following program: 98°C for 30 s, followed by 27 cycles of 98°C for 10 s, 55°C for 15 s, 68°C for 1 m, and a final extension period of 68°C for 1 m. Amplified DNA was electrophoresed on 2% agarose gel and stained with ethidium bromide.

**Protein extraction and analysis by SDS-PAGE followed by immunoblotting**

Soluble endosperm proteins were extracted from developing caryopses (12 to 15 DAF), whose embryo was removed, with 5 volumes (w/v) of extraction buffer containing 50 mM HEPES-NaOH (pH7.4), 2 mM MgCl_2_, 50 mM 2-mercaptoethanol, and 12.5% (v/v) glycerol. Homogenate was centrifuged twice at 20,000 *g* for 10 m at 4°C. The protein concentration of the supernatant was measured using Bio-Rad Protein Assay Dye Reagent Concentrate (Bio-Rad, Hercules, CA) with bovine serum albumin as a standard.

Ten micrograms of protein extract was denatured, separated on an 8% SDS-polyacrylamide gel (acrylamide: bisacrylamide = 40: 1), and transferred onto Immuno-Blot PVDF membrane (Bio-Rad). The blot was incubated with rabbit polyclonal antibody as described below followed by the alkaline phosphatase-conjugated secondary antibody. Signals were visualized with AP Conjugate Substrate Kit (Bio-Rad). Total proteins were stained with Coomassie Brilliant Blue R-250 for the loading control.

Soluble proteins and proteins loosely and tightly bound to starch were extracted from developing caryopses (12 to 15 DAF) as described by Asai *et al*. (2014). SDS-PAGE and immunoblotting were performed as above.

For quantification of protein signals, images were analyzed with ImageJ software (https://imagej.net/).

**Preparation of polyclonal antibody**

Polyclonal rabbit antisera targeted to rice starch biosynthetic enzymes were raised against synthetic peptides and purified by GenScript (http://www.genscript.com/) (Nanjing, China). The sequences of the peptide used were listed in Table S4.

Specific cross-reaction of anti-BEIIb antibody was confirmed by recognition of recombinant His-tagged mature BEIIb protein and disappearance of BEIIb-specific signal in soluble endosperm extract of EM10. Anti-SSI and -SSIIa antibodies specifically cross-reacted with His-tagged recombinant mature SSI and SSIIa, respectively. Pre-immune sera for anti-BEI, -BEIIa, -Pho1, and -PUL antibodies were used as negative controls and no cross-reaction was detected.

Anti-GBSSI antibody was provided by Dr. Naoko Fujita in Akita Prefectural University.

**Native-PAGE and activity staining**

Native-PAGE and activity staining of BE, SS, and DBE were performed according to Abe *et al*. (2014) with slight modifications. Twenty micrograms of soluble protein extract was separated on an 7.5% polyacrylamide gel (acrylamide: bisacrylamide = 29: 1), which contains 0.8% oyster glycogen (G8751, Sigma, Saint Louis, MO) and 0.3% potato tuber amylopectin (G8515, Sigma) for SS and DBE staining, respectively, at 4°C. For BE staining, after electrophoresis, the gel was washed once for 5 m with 50 mM HEPES-NaOH (pH7.4) and 10% (v/v) glycerol, and then incubated with reaction buffer containing 50 mM HEPES-NaOH (pH7.4), 2.5 mM AMP, 50 mM glucose-1-phosphate, 10% (v/v) glycerol, and 50 U rabbit muscle phosphorylase a (Sigma) for 15 h at room temperature. For SS staining, the gel was washed and incubated with buffers as described by Nishi *et al*. (2001) for 15 h at room temperature. For DBE staining, the gel was washed and incubated with buffers as described by Fujita *et al*. (1999) for 2 h at room temperature. After incubation, the gels were stained with 0.1% (w/v) I_2_ and 1% (w/v) KI until activity bands were visualized.

**Pyramiding mutant alleles using DNA markers**

Genomic DNA was extracted from leaf blade by shaking with 0.5 M NaOH and 0.2% (w/v) SDS. After centrifugation at 5,800 *g* for 3 m, an aliquot of supernatant was mixed with 100 volumes of 0.1 M Tris-HCl (pH8.0) and 1 mM EDTA. For SNP markers for detection of *age1* allele or *SSIIa^indica^* allele, PCR reaction mixtures (total 10 μl) containing 1 x reaction buffer, 0.2 mM dNTP mixture, 0.5 μl of genomic DNA, 2.5 pmol each of forward and reverse primers (Figure 6a), and 0.25 U of Takara Taq Hot Start Version (Takara-Bio) were used for PCR with the following program: 95°C for 1 m, followed by 33 or 35 cycles of 95°C for 30 s, 60°C for 30 s, 72°C for 30 s or 1 m. For detection of *age2* allele, reaction mixtures (total 10 μl) containing 1 x reaction buffer, 0.2 mM dNTP mixture, 0.5 μl of genomic DNA, 2 pmol each of forward and reverse primers (Figure 6a), and 0.125 U of PrimeSTAR GXL DNA Polymerase (Takara-Bio) were used for PCR as follows: 98°C for 30 s, followed by 30 cycles of 98°C for 10 s, 55°C for 15 s, 68°C for 1 m, and a final extension period of 68°C for 1 m.

**Determination of apparent amylose content**

Apparent amylose content was measured by an iodine colorimetric method as described previously (Yamakawa *et al.*, 2007). Values are shown as means ± SD of three biological replicates.

**Measurement of thermal properties of purified starch**

Rice flour was prepared from polished rice grains using a cyclone mill (CSM-S1, Fujiwara, Tokyo, Japan) equipped with a sieve screen of 0.25 mm diameter pore size and incubated with 0.05% (w/v) NaOH for 3 h at 4°C with gentle agitation. After centrifugation at 500 *g* for 3 m at 4°C, supernatant was removed. The pellet was washed 9 times with distilled water and then 3 times with 100% ethanol. The purified starch pellet was dried under vacuum overnight.

Thermal gelatinization properties of the starch were measured using a differential scanning calorimeter (DSC-60, Shimadzu, Kyoto, Japan). Purified starch (5 to 10 mg) was mixed with 4 volumes (w/v) of distilled water and 20 μl of the mixture was loaded onto the DSC pan. The sealed pan was heated from 35 to 100°C at a rate of 5°C per m. Values are shown as means ± SD of three biological replicates.

**Chain length distribution of amylopectin**

Samples for analysis of chain length distribution of amylopectin were prepared as described previously (Yamakawa *et al.*, 2007) and analyzed using a Dionex ICS-5000+ system (Thermo Fisher Scientific, Waltham, MA).

***In vitro* digestion property of purified starch by α-amylase**

Digestibility of starches was analyzed according the method of Kubo *et al*. (2010) with the following modification. Seven milligrams of purified starch, as described above, with 700 μl of reaction buffer containing 1 mM sodium glycerophosphate-HCl (pH6.9), 25 mM NaCl, and 5 mM CaCl_2_ was used as raw starch sample. The raw starch was boiled for 30 m to prepare gelatinized starch sample and then the gelatinized starch was stored at 4°C for 3 d to prepare retrograded starch sample. The starch samples were digested by adding 0.5 U of α-amylase from porcine pancreas (A3176, Sigma) at 37°C. Fifty microliter aliquots were drawn at different time intervals and immediately heated at 98°C for 10 m to inactivate α-amylase. The equivalent reducing sugar value of maltose was measured by the Somogyi-Nelson method (Nelson, 1944). Each sample was analyzed in triplicate.

**Sensory evaluation test of cooked rice**

Sensory evaluation test was performed using randomized combinations of Nipponbare, *age1*, *age2*, SSIIa-NIL, SSIIa/*age1*, and SSIIa/*age2*. Nipponbare, Toyonishiki, and Koshihikari cultivars were used as standards for intermediate (overall score = 0), bad (overall score = -2), and good (overall score = +2) palatability, respectively. Samples were tested in warm or cool conditions, in which cooked rice was cooled for 10 m or 2 h, respectively, after cooking at room temperature. At least 24 panelists evaluated these samples and compared them with the standard samples. The scores were rated between -5 (extremely bad) and +5 (extremely good). The test was done twice, in 2012 and 2015, at Hokuriku Research Station, Central Region Agricultural Research Center with a similar tendency and the results of 2015 were shown.

**References**

Abe, N., Asai, H., Yago, H., Oitome, N.F., Itoh, R., Crofts, N., Nakamura, Y. and Fujita, N. (2014) Relationships between starch synthase I and branching enzyme isozymes determined using double mutant rice lines. *BMC Plant Biol.* **14**, 80.

Asai, H., Abe, N., Matsushima, R., Crofts, N., Oitome, N.F., Nakamura, Y. and Fujita, N. (2014) Deficiencies in both starch synthase IIIa and branching enzyme IIb lead to a significant increase in amylose in SSIIa-inactive japonica rice seeds. *J. Exp. Bot.* **65**, 5497-5507.

Fujita, N., Kubo, A., Francisco, P.B., Nakakita, M., Harada, K., Minaka, N. and Nakamura, Y. (1999) Purification, characterization, and cDNA structure of isoamylase from developing endosperm of rice. *Planta* **208**, 283-293.

Hirochika, H. (2001) Contribution of the *Tos17* retrotransposon to rice functional genomics. *Curr. Opin. Plant Biol.* **4**, 118-122.

Kosugi, S., Natsume, S., Yoshida, K., MacLean, D., Cano, L., Kamoun, S. and Terauchi, R. (2013) Coval: Improving alignment quality and variant calling accuracy for next-generation sequencing data. *PLoS ONE* **8**, e75402.

Kubo, A., Akdogan, G., Nakaya, M., Shojo, A., Suzuki, S., Satoh, H. and Kitamura, S. (2010) Structure, physical, and digestive properties of starch from *wx ae* double-mutant rice. *J. Agric. Food Chem.* **58**, 4463-4469.

Kuroda, M., Kimizu, M. and Mikami, C. (2010) A simple set of plasmids for the production of transgenic plants. *Biosci. Biotechnol. Biochem.* **74**, 2348-2351.

Li, H. and Durbin, R. (2009) Fast and accurate short read alignment with Burrows-Wheeler transform. *Bioinformatics* **25**, 1754-1760.

Li, H., Handsaker, B., Wysoker, A., Fennell, T., Ruan, J., Homer, N., Marth, G., Abecasis, G., Durbin, R. and Genome Project Data, P. (2009) The sequence alignment/map format and SAMtools. *Bioinformatics* **25**, 2078-2079.

Miyao, A., Tanaka, K., Murata, K., Sawaki, H., Takeda, S., Abe, K., Shinozuka, Y., Onosato, K. and Hirochika, H. (2003) Target site specificity of the *Tos17* retrotransposon shows a preference for insertion within genes and against insertion in retrotransposon-rich regions of the genome. *Plant Cell* **15**, 1771-1780.

Nakagawa, T., Kurose, T., Hino, T., Tanaka, K., Kawamukai, M., Niwa, Y., Toyooka, K., Matsuoka, K., Jinbo, T. and Kimura, T. (2007) Development of series of gateway binary vectors, pGWBs, for realizing efficient construction of fusion genes for plant transformation. *J. Biosci. Bioeng.* **104**, 34-41.

Nelson, N. (1944) A photometric adaptation of the Somogyi method for the determination of glucose. *J. Biol. Chem.* **153**, 375-380.

Nishi, A., Nakamura, Y., Tanaka, N. and Satoh, H. (2001) Biochemical and genetic analysis of the effects of *amylose-extender* mutation in rice endosperm. *Plant Physiol.* **127**, 459-472.

Toki, S. (1997) Rapid and efficient *Agrobacterium*-mediated transformation in rice. *Plant Mol. Biol. Rep.* **15**, 16-21.

Yamakawa, H., Hirose, T., Kuroda, M. and Yamaguchi, T. (2007) Comprehensive expression profiling of rice grain filling-related genes under high temperature using DNA microarray. *Plant Physiol.* **144**, 258-277.

Yamamoto, T., Nagasaki, H., Yonemaru, J., Ebana, K., Nakajima, M., Shibaya, T. and Yano, M. (2010) Fine definition of the pedigree haplotypes of closely related rice cultivars by means of genome-wide discovery of single-nucleotide polymorphisms. *BMC Genomics* **11**, 267.
